# Response of sea surface temperature to atmospheric rivers

**DOI:** 10.1038/s41467-024-48486-9

**Published:** 2024-06-12

**Authors:** Tien-Yiao Hsu, Matthew R. Mazloff, Sarah T. Gille, Mara A. Freilich, Rui Sun, Bruce D. Cornuelle

**Affiliations:** 1 Scripps Institution of Oceanography, University of California San Diego, La Jolla, 92093 CA USA; 2 Brown University, Department of Earth, Environmental and Planetary Sciences and Division of Applied Mathematics, Providence, 02912 RI USA

**Keywords:** Physical oceanography, Atmospheric science

## Abstract

Atmospheric rivers (ARs), responsible for extreme weather conditions, are mid-latitude systems that can cause significant damage to coastal areas. While forecasting ARs beyond two weeks remains a challenge, past research suggests potential benefits may come from properly accounting for the changes in sea surface temperature (SST) through air–sea interactions. In this paper, we investigate the impact of ARs on SST over the North Pacific by analyzing 25 years of ocean reanalysis data using an SST budget equation. We show that in the region of strong ocean modification, ocean dynamics can offset over 100% of the anomalous SST warming that would otherwise arise from atmospheric forcing. Among all ocean processes, ageostrophic advection and vertical mixing (diffusion and entrainment) are the most important factors in modifying the SST tendency response. The SST tendency response to ARs varies spatially. For example, in coastal California, the driver of enhanced SST warming is the reduction in ageostrophic advection due to anomalous southerly winds. Moreover, there is a large region where the SST shows a warming response to ARs due to the overall reduction in the total clouds and subsequent increase in total incoming shortwave radiation.

## Abstract (web summary)

Strong air-sea interactions during atmospheric rivers often lead to modest upper ocean heat changes. The authors show that interior ocean dynamics are compensating for these air-sea exchanges. These findings can help improve subseasonal forecasts.

## Introduction

Atmospheric rivers (ARs), characterized by moisture filaments extending thousands of kilometers from the tropics, are responsible for over 90% of meridional water vapor transport from low to mid-latitudes^1,2^. These events have profound impacts on coastal regions. In California, ARs are responsible for up to 50% of annual rainfall, and 40% to 90% of them are associated with major floods^1,3,4^, causing more than one billion USD in damage annually^5^. Past studies have identified predictors of AR activity, such as El Niño Southern Oscillation (ENSO) and Madden–Julian Oscillation^6–8^. However, the predictability of ARs does not exceed 10–20 days depending on the metric chosen^9–11^. An important factor contributing to the uncertainty of AR predictions is likely feedbacks associated with their modification of the local sea surface boundary conditions through air–sea interactions^12^.

The extremes associated with ARs include strong wind, high moisture, intense precipitation, and warm air temperature^13^. In the upper ocean, ARs perturb sea surface temperature (SST), sea surface height, sea surface current, and mixed layer depth (MLD). Qualitatively, the physical expectation is that a relatively moist and warm AR heats the surface ocean through increasing downward heat fluxes. Simultaneously, its wind enhances vertical mixing at the base of the mixed layer that cools the surface ocean. ARs’ general impact was first studied by Shinoda et al.^14^ using five years of composited ARs. They found that ocean entrainment is important in SST cooling, extending downstream from the AR center. In parallel, other evidence shows that air–sea interaction under long-duration AR events can have a signature on synoptic timescales. For example, Sun et al.^12^ showed that when fixed-SST boundary conditions are replaced with an interactive ocean model, the prediction skill of the integrated water vapor (IWV) improves by 12%, and the prediction skill of integrated vapor transport (IVT) improves by 6% for events associated with stronger cooling periods. However, the studies mentioned above have not quantified the response of SST to ARs in terms of each atmospheric and oceanic process, nor have the studies identified AR spatial dependency, leaving a gap in our understanding of AR impacts on the ocean. This study addresses this gap by using an ocean state estimate with an SST budget equation to diagnose the specific physical processes that govern the SST tendency response to ARs.

## Results

In this paper, we focus on Oct–Mar when ARs are the most active in the North Pacific. First, we will show ARs’ general characteristics of water years during 1993–2017 (water year *n* starts Oct 1 of year *n*−1). Then, we analyze the SST tendency response to ARs over the North Pacific. Finally, we present the spatial and temporal variability of SST tendency to ARs. We have removed the 15-day-smoothed daily climatology of all variables that will be shown, such that they are all anomalies. Ocean data are obtained from Estimating the Circulation and Climate of the Ocean Version 4 release 4 (ECCOv4)^15^, and atmospheric data are obtained from ECMWF Reanalysis - Interim (ERA-Interim)^16^ (see Methods for details).

### Climatology of ARs and associated atmospheric forcing

**Fig. 1:**
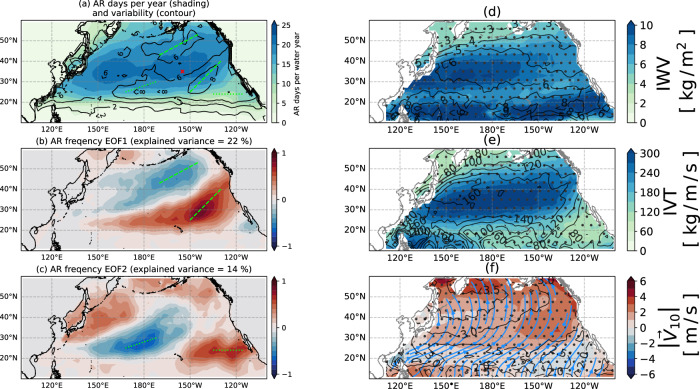
Atmospheric river (AR) characteristics during water years 1993–2017. **a** The averaged number of AR days in a water year during Oct–Mar (shading) and the standard deviation as interannual variability (contours). Each water year is treated as one sample, meaning the statistics are constructed from 25 samples. The green dashed and dotted lines mark the peaks of the interannual variability that match the peaks of each EOF. The red cross marks the location where the sea surface temperature tendency decomposition is shown in Fig. 4. **b**, **c** The first two empirical orthogonal functions (EOFs) of the averaged AR days in a water year (shading). **d** Composite integrated water vapor (IWV) anomaly during AR days (shading) and its standard deviation (contours). Each AR day is treated as one sample. **e** Same as **d** but compositing integrated vapor transport (IVT) anomaly. **f** Same as **d** but compositing 10-m wind anomaly, with magnitudes of the anomaly indicated by shading and direction by blue streamlines. **d**–**f** Dotted areas show response regions that pass the significance test (*p* = 0.05).

The mean number of AR days during Oct–Mar peaks along latitude 35^°^N, as shown in Fig. 1a. The interannual variability is concentrated along two pairs of ridges, labeled by dashed and dotted light green lines in Fig. 1a. These two pairs of variability ridges are associated with two leading empirical orthogonal functions (EOFs; shown in Fig. 1b, c. EOF1 and EOF2 roughly represent the shift of AR activity from south to north and west to east, respectively. EOF1 positively correlates with the NINO3.4 SST index (95% confidence interval). This connection arises because ENSO events tend to deepen the Aleutian Low, which controls the location of stormtrack activity^17–20^. This result is consistent with Tseng et al.^6^, who examined this relationship more closely. EOF2 positively correlates with NINO3.4 SST index (80% confidence interval) as well, although the correlation is weaker. These results indicate that ENSO can change ARs’ north-south and east-west frequency. A deeper physical understanding is beyond the scope of this paper and will require future investigations.

To isolate AR impact on SST tendencies, we remove the daily climatology (15-day smoothed) and show only the anomalous component. The composited AR surface forcing is shown in Fig. 1d–f. ARs carry anomalous high IWV and IVT. The anomalous 10-m winds that ARs bring have a latitudinal dependency. Southward of 45°N, ARs accompany anomalous westerly wind, while northward of 45°N, they carry anomalous easterlies. This is consistent with the idea that typical ARs are generated by extending moisture filaments from low latitudes at the southeastern flank of mid-latitude cyclones, and the moisture transport turns northwestward as the counterclockwise airflow advects it^14^. Northward of 30^°^N the 10-m wind speed increases, whereas southward of 30^°^N there is a slight decrease of wind because the anomalous wind opposes the trade wind.

### How do ARs modulate SST?

Using the mean mixed layer potential temperature, $\overline{\Theta }$, as a proxy for SST in the SST budget equation, we can decompose the local SST tendency, ${\dot{\overline{\Theta }}}_{{{{{{{{\rm{loc}}}}}}}}}=\partial \overline{\Theta }/\partial t$, into the contributions from shortwave radiation, longwave radiation, sensible heat flux, latent heat flux, dilution effect (rain that enters the surface ocean directly changes the SST), residual velocity (sum of Eulerian and bolus velocity) advection, vertical mixing (diffusion and entrainment), detrainment, and horizontal diffusion. Formally, the tendency is written as

1 $${\dot{\overline{\Theta }}}_{{{{{{{{\rm{loc}}}}}}}}}=\overbrace{{\dot{\overline{\Theta }}}_{{{{{{{{\rm{sw}}}}}}}}}+{\dot{\overline{\Theta }}}_{{{{{{{{\rm{lw}}}}}}}}}+{\dot{\overline{\Theta }}}_{{{{{{{{\rm{sen}}}}}}}}}+{\dot{\overline{\Theta }}}_{{{{{{{{\rm{lat}}}}}}}}}+{\dot{\overline{\Theta }}}_{{{{{{{{\rm{dilu}}}}}}}}}}^{\begin{array}{c}{\dot{\overline{\Theta }}}_{{{{{{{{\rm{sfc}}}}}}}}}\end{array}}+\overbrace{{\dot{\overline{\Theta }}}_{{{{{{{{\rm{adv}}}}}}}}}+{\dot{\overline{\Theta }}}_{{{{{{{{\rm{vmix}}}}}}}}}+{\dot{\overline{\Theta }}}_{\det }+{\dot{\overline{\Theta }}}_{{{{{{{{\rm{hdif}}}}}}}}}}^{\begin{array}{c}{\dot{\overline{\Theta }}}_{{{{{{{{\rm{ocn}}}}}}}}}\end{array}}.$$

We group terms into the contribution from the surface forcing, ${\dot{\overline{\Theta }}}_{{{{{{{{\rm{sfc}}}}}}}}}$, and the ocean response, ${\dot{\overline{\Theta }}}_{{{{{{{{\rm{ocn}}}}}}}}}$, as indicated by the overbraces. The detailed definition of each term is documented in the Methods Section.

**Fig. 2:**
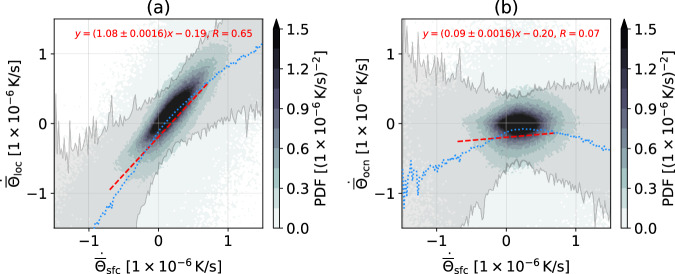
Histogram of occurrence of the sea surface temperature tendencies in the extratropical North Pacific (northward of 30^°^N). **a** Anomalous ${\dot{\overline{\Theta }}}_{{{{{{{{\rm{sfc}}}}}}}}}$ versus anomalous ${\dot{\overline{\Theta }}}_{{{{{{{{\rm{loc}}}}}}}}}$. **b** Anomalous ${\dot{\overline{\Theta }}}_{{{{{{{{\rm{sfc}}}}}}}}}$ versus anomalous ${\dot{\overline{\Theta }}}_{{{{{{{{\rm{ocn}}}}}}}}}$. Each axis has 100 bins between $\left[-1.5\times 1{0}^{-6},1.5\times 1{0}^{-6}\right]\,{{{{{{{\rm{K}}}}}}}}/{{{{{{{\rm{s}}}}}}}}$. The red dashed lines are the linear regressions of each distribution with their equations printed in red text. The blue dotted lines and gray shading are the mean and spanned one standard deviation, respectively, of the data binned according to ${\dot{\overline{\Theta }}}_{{{{{{{{\rm{sfc}}}}}}}}}$. The bin interval is 2 × 10^−8^ K s^−1^.

We first want to show that ocean dynamics modulate the anomalous SST tendency. Figure 2a shows the relationship between the anomalous ${\dot{\overline{\Theta }}}_{{{{{{{{\rm{sfc}}}}}}}}}$ and anomalous ${\dot{\overline{\Theta }}}_{{{{{{{{\rm{loc}}}}}}}}}$. The slope deviates by 8% from the line ${\dot{\overline{\Theta }}}_{{{{{{{{\rm{loc}}}}}}}}}={\dot{\overline{\Theta }}}_{{{{{{{{\rm{sfc}}}}}}}}}$, meaning ${\dot{\overline{\Theta }}}_{{{{{{{{\rm{sfc}}}}}}}}}$ itself cannot fully explain the ${\dot{\overline{\Theta }}}_{{{{{{{{\rm{loc}}}}}}}}}$. Also, anomalous ${\dot{\overline{\Theta }}}_{{{{{{{{\rm{sfc}}}}}}}}}=0$ implies negative anomalous ${\dot{\overline{\Theta }}}_{{{{{{{{\rm{loc}}}}}}}}}$. Together, these results suggest that other factors, such as wind-induced mixing, might be important, and further investigation is necessary. In Fig. 2b we plot the joint probability distribution between ${\dot{\overline{\Theta }}}_{{{{{{{{\rm{sfc}}}}}}}}}$ and ${\dot{\overline{\Theta }}}_{{{{{{{{\rm{ocn}}}}}}}}}$ anomalies, effectively removing the slope 1 in 2a. Since the relationship between ${\dot{\overline{\Theta }}}_{{{{{{{{\rm{sfc}}}}}}}}}$ and ${\dot{\overline{\Theta }}}_{{{{{{{{\rm{ocn}}}}}}}}}$ anomalies is not linear (correlation is only 0.07), we plot the mean and standard error with dotted blue lines and gray shading, respectively, to view ${\dot{\overline{\Theta }}}_{{{{{{{{\rm{ocn}}}}}}}}}$ as a nonlinear function of ${\dot{\overline{\Theta }}}_{{{{{{{{\rm{sfc}}}}}}}}}$. Two distinct regimes emerge. The first one is ${\dot{\overline{\Theta }}}_{{{{{{{{\rm{sfc}}}}}}}}}$ roughly above 0.2 × 10^−6^ K/s, where more positive ${\dot{\overline{\Theta }}}_{{{{{{{{\rm{sfc}}}}}}}}}$ induces more negative ${\dot{\overline{\Theta }}}_{{{{{{{{\rm{ocn}}}}}}}}}$, meaning the ocean is opposing the warming forced by the surface fluxes. When ${\dot{\overline{\Theta }}}_{{{{{{{{\rm{sfc}}}}}}}}}$ is below roughly 0.2 × 10^−6^ K/s, more negative ${\dot{\overline{\Theta }}}_{{{{{{{{\rm{sfc}}}}}}}}}$ induces more negative ${\dot{\overline{\Theta }}}_{{{{{{{{\rm{ocn}}}}}}}}}$, implying that the ocean amplifies the cooling forced by surface fluxes.

**Fig. 3:**
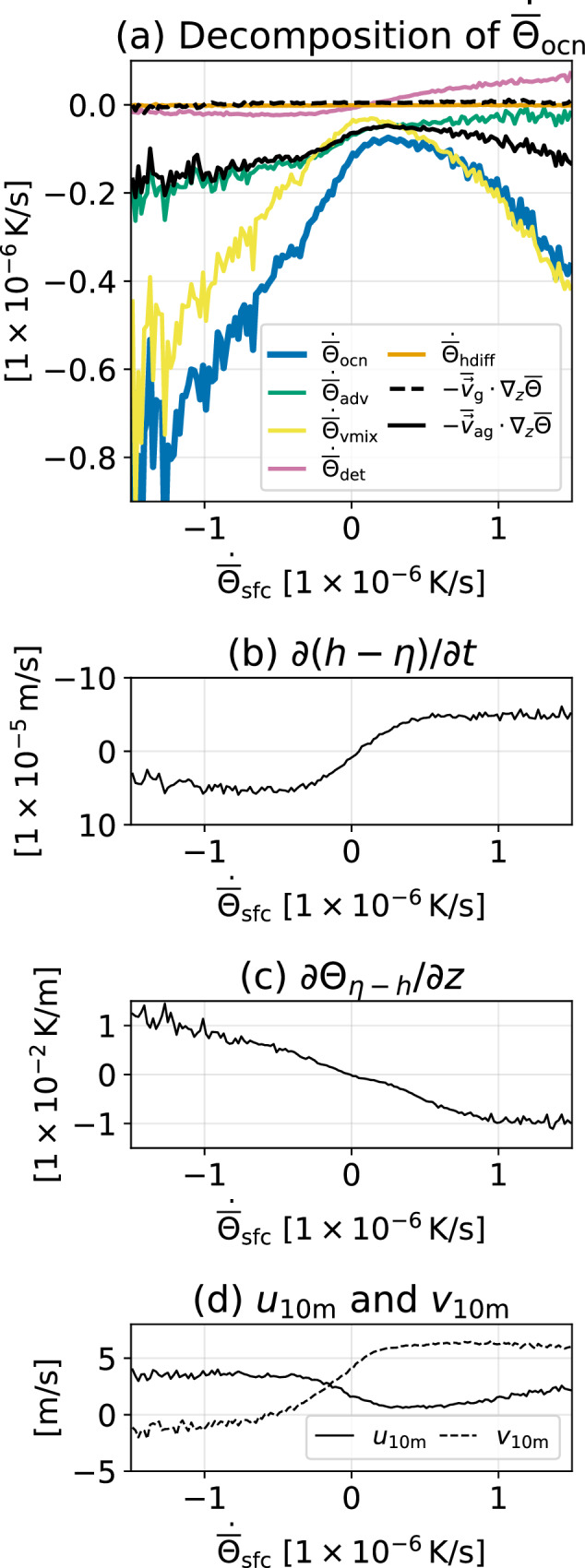
Analysis to understand the mean anomalous sea surface temperature tendency due to ocean process ${\dot{\overline{\Theta }}}_{{{{{{{{\rm{ocn}}}}}}}}}$. **a** Decomposition of ${\dot{\overline{\Theta }}}_{{{{{{{{\rm{ocn}}}}}}}}}$ as a function of surface fluxes ${\dot{\overline{\Theta }}}_{{{{{{{{\rm{sfc}}}}}}}}}$. **b** the same as **a** but for anomalous entrainment speed $\partial \left(h-\eta \right)/\partial t$. **c** the same as **a** but for the vertical temperature gradient at the bottom of the mixed layer (*z* = *η* − *h*). **d** the same as **a** but for the zonal (*u*_10m_, solid line) and meridional (*v*_10m_, dashed line) 10-m winds. Each curve connects the averages of the binned data. The bin interval is 2 × 10^−8^ K s^−1^.

We can break down ${\dot{\overline{\Theta }}}_{{{{{{{{\rm{ocn}}}}}}}}}$ into ocean processes as shown in Fig. 3a by using the decomposition Equation (1). When the surface forcing anomalously cools the SST, ${\dot{\overline{\Theta }}}_{{{{{{{{\rm{sfc}}}}}}}}} < 0$, the mixed layer deepens anomalously as shown in the anomalous $\partial \left(h-\eta \right)/\partial t\approx \partial h/\partial t$ in Fig. 3b (*h* is the mixed layer thickness, and *η* is the surface height anomaly from the mean ocean depth). Usually, mixed layer deepening induces SST cooling, with exceptions occurring in barrier layers when salinity dominates the stratification such that there is little temperature gradient at the base of the mixed layer. As the mixed layer deepens, the even cooler deeper ocean water results in a larger vertical temperature gradient (Fig. 3c), therefore producing stronger vertical mixing cooling. When the surface forcing anomalously warms the SST, ${\dot{\overline{\Theta }}}_{{{{{{{{\rm{sfc}}}}}}}}} > 0$, the mixed layer anomalously shoals. The shoaling induces detrainment that leads to an increase in the vertically averaged mixed layer potential temperature $\overline{\Theta }$. Since detrainment does not change SST, the increase is because the SST, slightly warmer than the water below, has a greater impact when computing $\overline{\Theta }$ with shallower MLD. The anomalously reduced MLD also results in a weaker vertical temperature gradient at the base of the mixed layer ∂Θ_*η*−*h*_/∂*z* (Fig. 3c). However, weaker ∂Θ_*η*−*h*_/∂*z* does not lead to weaker vertical mixing ${\dot{\overline{\Theta }}}_{{{{{{{{\rm{vmix}}}}}}}}}$ (Fig. 3a). This shows that the turbulent wind work is strong such that the turbulent kinetic energy in the oceanic boundary layer remains vigorous. Therefore, the downward transport of the turbulent kinetic energy extends the high vertical diffusivity *K*_*V*_ out of the shoaled mixed layer and maintains a strong SST cooling. This is consistent with the fact the cooling of ${\dot{\overline{\Theta }}}_{{{{{{{{\rm{vmix}}}}}}}}}$ grows linearly as ${\dot{\overline{\Theta }}}_{{{{{{{{\rm{sfc}}}}}}}}}$ increase. Even when ${\dot{\overline{\Theta }}}_{{{{{{{{\rm{sfc}}}}}}}}}$ is zero, anomalous vertical mixing ${\dot{\overline{\Theta }}}_{{{{{{{{\rm{vmix}}}}}}}}}$ can still happen due to the increased anomalous wind (Fig. 3d) that generates turbulent kinetic energy and thus higher *K*_*V*_.

The anomalous advection ${\dot{\overline{\Theta }}}_{{{{{{{{\rm{adv}}}}}}}}}$ produces strongest cooling when the anomalous ${\dot{\overline{\Theta }}}_{{{{{{{{\rm{sfc}}}}}}}}}$ is near zero, where ${\dot{\overline{\Theta }}}_{{{{{{{{\rm{adv}}}}}}}}}$ is dominated by ageostrophic advection. The ageostrophic contribution here can be attributed to Ekman advection because its cooling strength aligns with the anomalous zonal 10-m wind *u*_10m_ (solid line in Fig. 3d). For large positive anomalous ${\dot{\overline{\Theta }}}_{{{{{{{{\rm{sfc}}}}}}}}} > 0.5\times 1{0}^{-6}{{{{{{{\rm{K}}}}}}}}/{{{{{{{\rm{s}}}}}}}}$, the cooling due to anomalous ageostrophic advection $-{\overline{{{{{{{{\bf{v}}}}}}}}}}_{{{{{{{{\rm{ag}}}}}}}}}\cdot {\nabla } \, _{z}\overline{\Theta }$ is strong while the overall anomalous cooling due to advection ${\dot{\overline{\Theta }}}_{{{{{{{{\rm{adv}}}}}}}}}$ is weak. This is a consequence of the cancellation of Ekman advection cooling by the bolus velocity-driven advection, eddy, and entrainment as detailed in Equation (13) (see Section Methods). This strong anomalous ${\dot{\overline{\Theta }}}_{{{{{{{{\rm{sfc}}}}}}}}}$ is located in the strong marine warming region $\left[1{5}^{\circ }\;{{{{{{{\rm{N}}}}}}}},2{5}^{\circ }\;{{{{{{{\rm{N}}}}}}}}\right]\times \left[12{0}^{\circ }\;{{{{{{{\rm{W}}}}}}}},13{5}^{\circ }\;{{{{{{{\rm{W}}}}}}}}\right]$. As we will see later, this region has a very different SST tendency response due to the pre-existing stratocumulus clouds.

**Fig. 4:**
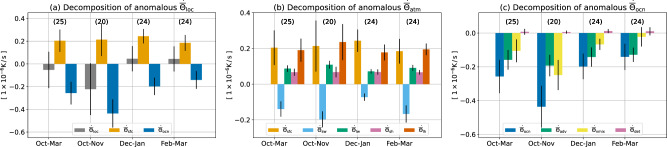
The sea surface temperature (SST) tendency analysis at the example atmospheric river (AR) active location $\left(3{5}^{\circ }{{{{{{{\rm{N}}}}}}}},15{1}^{\circ }{{{{{{{\rm{W}}}}}}}}\right)$ labeled as the red cross in Fig. 1a. **a** Anomalous local SST tendency ${\dot{\overline{\Theta }}}_{{{{{{{{\rm{loc}}}}}}}}}$, SST tendency due to surface fluxes ${\dot{\overline{\Theta }}}_{{{{{{{{\rm{atm}}}}}}}}}$, and SST tendency due to ocean modification ${\dot{\overline{\Theta }}}_{{{{{{{{\rm{ocn}}}}}}}}}$. The decomposition is defined in Equation (17). **b** Anomalous ${\dot{\overline{\Theta }}}_{{{{{{{{\rm{atm}}}}}}}}}$ and its decomposition as in Equation (15). **c** Anomlaous ${\dot{\overline{\Theta }}}_{{{{{{{{\rm{ocn}}}}}}}}}$ and its decomposition as in Equation (16). The bars and whiskers show the mean values and one standard deviation of the grouped data, and the numbers in parenthesis show the number of valid years used to do the calculation. We first compute the mean and standard deviation of a specific time range (Oct–Mar, Dec–Jan, etc) of each year. We discard years with fewer than 5 AR days within the selected months. Then, the derived mean values of each valid year are used to compute the average of means and standard error, i.e., the confidence of the estimate.

We now want to better understand AR forcing over the AR-active region, where ocean dynamics can have a large impact. In Fig. 4, we plot a bar chart of SST tendency decomposition at $\left[3{5}^{\circ }\;{{{{{{{\rm{N}}}}}}}},15{1}^{\circ }\;{{{{{{{\rm{W}}}}}}}}\right]$, marked with a red cross in Fig. 1a. ARs cause strong anomalous SST warming, and ocean dynamics overall offset over 100% of this anomalous warming that would have otherwise increased SST (Fig. 4a). Warming due to surface forcing comes from reduced evaporation, surface sensible heat loss, and longwave radiation (Fig. 4b). On the other hand, cooling is due to reduced incoming shortwave radiation (Fig. 4b). These anomalous forcing terms are opposite in sign to their climatological values (Fig. S1b). In the ocean, anomalous cooling is due to cold advection and vertical mixing (Fig. 4c).

Finally, the anomalous SST tendency response has seasonality, which can be due to the seasonality of the MLD. Since the MLD is shallower in October and reaches its maximum by March, the anomalous SST tendencies, ${\dot{\overline{\Theta }}}_{{{{{{{{\rm{sfc}}}}}}}}}$ and ${\dot{\overline{\Theta }}}_{{{{{{{{\rm{ocn}}}}}}}}}$, are generally the strongest in October and weakest in March. In Feb–Mar, the strengths of detrainment ${\dot{\overline{\Theta }}}_{\det }$ and vertical mixing ${\dot{\overline{\Theta }}}_{{{{{{{{\rm{vmix}}}}}}}}}$ are comparable, and the anomalous SST tendency (Fig. 4a) is comparable to climatology (Fig. S1a). It shows that the role of air–sea interaction becomes important in early spring because the total surface heat flux is important in the evolution of MLD^21,22^. Overall, the strong cancellation between surface fluxes and ocean response makes the local SST tendency ${\dot{\overline{\Theta }}}_{{{{{{{{\rm{loc}}}}}}}}}$ a non-trivial function of time and space. Later, we will discuss the seasonality in greater detail.

### Spatial distribution of anomalous SST tendency response to ARs

**Fig. 5:**
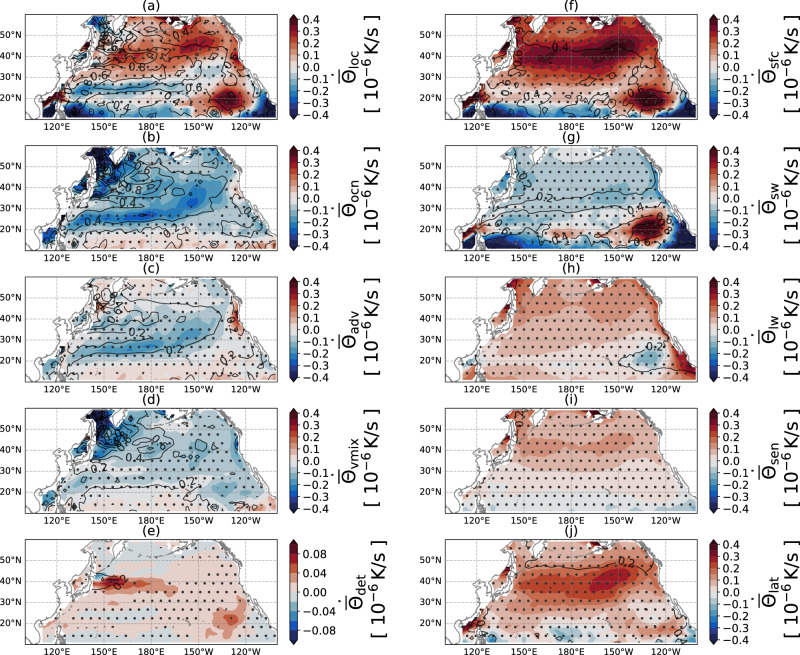
The response of sea surface temperature (SST) tendency to atmospheric rivers (ARs), decomposed into distinct contributing processes. Variables shown are: **a** ${\dot{\overline{\Theta }}}_{{{{{{{{\rm{loc}}}}}}}}}$, **b** ${\dot{\overline{\Theta }}}_{{{{{{{{\rm{ocn}}}}}}}}}$, **c** ${\dot{\overline{\Theta }}}_{{{{{{{{\rm{adv}}}}}}}}}$, **d** ${\dot{\overline{\Theta }}}_{{{{{{{{\rm{vmix}}}}}}}}}$, **e** ${\dot{\overline{\Theta }}}_{\det }$, **f** ${\dot{\overline{\Theta }}}_{{{{{{{{\rm{sfc}}}}}}}}}$, **g** ${\dot{\overline{\Theta }}}_{{{{{{{{\rm{sw}}}}}}}}}$, **h** ${\dot{\overline{\Theta }}}_{{{{{{{{\rm{lw}}}}}}}}}$, **i** ${\dot{\overline{\Theta }}}_{{{{{{{{\rm{sen}}}}}}}}}$, and **j** ${\dot{\overline{\Theta }}}_{{{{{{{{\rm{lat}}}}}}}}}$. Each panel shows the mean of the composite anomalous SST tendency associated with a particular process (shading) and its standard deviation (contours). Dotted areas show response regions that pass the significance test (*p* = 0.05). For each grid point, we compute the mean and standard deviation by grouping all of the AR day data. The significance test is tested against the climatology group, i.e., every single day. The anomalous SST tendencies ${\dot{\overline{\Theta }}}_{{{{{{{{\rm{dilu}}}}}}}}}$ and ${\dot{\overline{\Theta }}}_{{{{{{{{\rm{hdiff}}}}}}}}}$ are very small such that they are not shown.

**Fig. 6:**
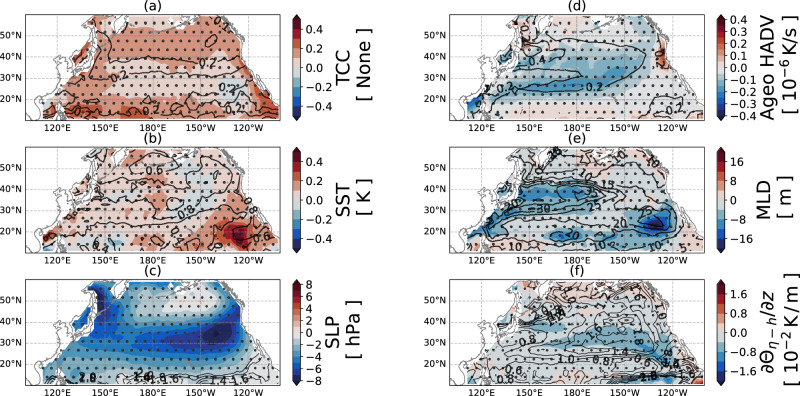
Auxiliary quantities to help understand the sea surface temperature (SST) response to ARs. **a** The anomalous total cloud cover (TCC) in fraction. **b** The anomalous SST. **c** The anomalous sea level pressure (SLP). **d** The anomalous SST tendency due to ageostrophic advection. **e** The anomalous mixed layer depth (MLD). **f** The anomalous vertical temperature gradient at the base of mixed layer ∂Θ_*η*−*h*_/∂*z*. Each panel shows the mean of the composited variable (shading) and its standard deviation (contours). The hatches denote the region that passes the significant test (*p* = 0.05).

For a more comprehensive understanding, the analysis in the previous subsection can be extended to every 2° × 2° grid box over the North Pacific. Figure 5 shows the anomalous daily SST tendency response to ARs in the extratropical northern Pacific during Oct–Mar, with auxiliary variables shown in Fig. 6 to clarify the results. The local SST tendency response ${\dot{\overline{\Theta }}}_{{{{{{{{\rm{loc}}}}}}}}}$ (Fig. 5a) is decomposed into the contribution of surface forcing ${\dot{\overline{\Theta }}}_{{{{{{{{\rm{sfc}}}}}}}}}$ (Fig. 5f) and ocean dynamics ${\dot{\overline{\Theta }}}_{{{{{{{{\rm{ocn}}}}}}}}}$ (Fig. 5b). The local anomalous SST tendency ${\dot{\overline{\Theta }}}_{{{{{{{{\rm{loc}}}}}}}}}$ shows a warming response roughly northward of 30°N and cooling southward. This pattern is driven by the surface forcing ${\dot{\overline{\Theta }}}_{{{{{{{{\rm{sfc}}}}}}}}}$, while the oceanic response ${\dot{\overline{\Theta }}}_{{{{{{{{\rm{ocn}}}}}}}}}$ opposes it. The warming signal comes from the increased longwave radiation, sensible heat flux, and latent heat flux (Fig. 5h–j). Cooling is generated by a reduction in shortwave radiation forcing ${\dot{\overline{\Theta }}}_{{{{{{{{\rm{sw}}}}}}}}}$ (Fig. 5g) because of the increased total cloud cover that blocks sunlight (Fig. 6a). The response in the region $\left[12{0}^{\circ }\;{{{{{{{\rm{W}}}}}}}},13{5}^{\circ }\;{{{{{{{\rm{W}}}}}}}}\right]\times \left[1{5}^{\circ }\;{{{{{{{\rm{N}}}}}}}},2{5}^{\circ }\;{{{{{{{\rm{N}}}}}}}}\right]$ is different from other regions, as here the anomalous SST tendency exceeds 0.3 K/day. Henceforth, we refer to this as the strong marine warming region, which will be discussed separately below.

The ocean dynamics component ${\dot{\overline{\Theta }}}_{{{{{{{{\rm{ocn}}}}}}}}}$ (Fig. 5b) shows the strongest SST cooling along the northwestern coastal region, which is dominated by the vertical mixing ${\dot{\overline{\Theta }}}_{{{{{{{{\rm{vmix}}}}}}}}}$ (Fig. 5d). From 20°–50°N, ARs bring anomalous westerlies (Fig. 1f), which produce strong advective cooling ${\dot{\overline{\Theta }}}_{{{{{{{{\rm{adv}}}}}}}}}$ (Fig. 5c) that is mostly southward Ekman advection (Fig. 6d). In most of the domain, the increased heat fluxes outcompete the wind work and shoal the mixed layer (Fig. 6e). Therefore, they induce anomalous detrainment ${\dot{\overline{\Theta }}}_{\det }$ that (Fig. 5e) increases the mixed layer potential temperature $\overline{\Theta }$. The effect of detrainment is strongest along the Kuroshio Extension, a region rich in SST mesoscale eddies. We do not investigate further because ECCOv4’s 1° resolution is not ideal for understanding oceanic eddies. Another location with strong detrainment is the strong marine warming region, which will be discussed below.

Along the California coast, there is a strong anomalous warming response due to anomalous Ekman advection (Figs. 5c and 6d) driven by the anomalous southerly winds (Fig. 1f). This warming is opposed by anomalous vertical mixing ${\dot{\overline{\Theta }}}_{{{{{{{{\rm{vmix}}}}}}}}}$ (Fig. 5d). Because there is no notable change in MLD and ∂Θ_*η*−*h*_/∂*z* (Fig. 6e and f) and their patterns do not match ${\dot{\overline{\Theta }}}_{{{{{{{{\rm{vmix}}}}}}}}}$, we rule out the possibility of a change in vertical thermal structure. Rather, this evidence shows that there is generation of turbulent kinetic energy, associated with the anomalous Ekman advection, which is transported downward and extends high vertical diffusivity *K*_*V*_ beyond the mixed layer, therefore increasing the cooling due to vertical diffusion ${\dot{\overline{\Theta }}}_{{{{{{{{\rm{vmix}}}}}}}}}$.

The interannual variability of anomalous SST tendency response is mostly located at the Kuroshio Extension and in the strong marine warming region, indicated by the contours in Fig. 5a. The ocean response ${\dot{\overline{\Theta }}}_{{{{{{{{\rm{ocn}}}}}}}}}$ (Fig. 5b) contributes more variability in the Kuroshio Extension region than the surface fluxes ${\dot{\overline{\Theta }}}_{{{{{{{{\rm{sfc}}}}}}}}}$ (Fig. 5f), highlighting the role of mesoscale eddies as they enhance the air–sea fluxes when AR passes from above^23^. Along 40°N this variability is attributed to advection ${\dot{\overline{\Theta }}}_{{{{{{{{\rm{adv}}}}}}}}}$ and vertical mixing ${\dot{\overline{\Theta }}}_{{{{{{{{\rm{vmix}}}}}}}}}$ (Fig. 5c, d), whereas the contribution of vertical mixing is restricted to the Pacific Ocean near Japan.

### Seasonality of anomalous SST tendency response to ARs

While the qualitative roles of different processes do not change, the anomalous SST tendency has a notable seasonality. There is a strong cancellation between surface fluxes and ocean modification in Oct–Nov and Feb–Mar (Figs. S2 and S3). The warming of atmospheric forcing decays as the months progress. The location of the strongest warming follows the latitudinal position of the strongest meridional SST gradient, which is also the stormtrack location. The advective cooling ${\dot{\overline{\Theta }}}_{{{{{{{{\rm{adv}}}}}}}}}$ consequently shows a similar progression in time and latitudinal location (Fig. S3). In the meanwhile, cooling due to the vertical mixing ${\dot{\overline{\Theta }}}_{{{{{{{{\rm{vmix}}}}}}}}}$ is strong in Oct–Nov, but almost muted in Dec–Jan. This is because the mixed layer is seasonally thinner in fall and thicker in spring, and the vertical temperature gradient at the base of the mixed layer ∂Θ_*η*−*h*_/∂*z* is correspondingly stronger and weaker. Therefore, anomalous SST tendency due to vertical mixing ${\dot{\overline{\Theta }}}_{{{{{{{{\rm{vmix}}}}}}}}}$ is stronger in fall and weaker in later months (Fig. S4). On the other hand in Feb–Mar, there is a modest cooling due to vertical mixing. This is likely because in early spring, AR forcing is compatible with seasonal forcing such that AR-induced anomalous surface fluxes ${\dot{\overline{\Theta }}}_{{{{{{{{\rm{sfc}}}}}}}}}$ have stronger control over the MLD. Therefore, when an AR arrives, the warming can effectively reduce the MLD (Fig. S4) and in turn enhance cooling due to vertical mixing.

### Strong marine warming region over the East Pacific

The strong marine warming region $\left[1{5}^{\circ }\;{{{{{{{\rm{N}}}}}}}},2{5}^{\circ }\;{{{{{{{\rm{N}}}}}}}}\right]\times \left[12{0}^{\circ }\;{{{{{{{\rm{W}}}}}}}},13{5}^{\circ }\;{{{{{{{\rm{W}}}}}}}}\right]$ (Fig. 5a) responds differently from other parts of the ocean. In this region, AR conditions increase the shortwave radiation (Fig. 5g) and slightly decrease the longwave radiation (Fig. 5h), which together warm the SST and shoal the MLD (Fig. 6e). The shoaling of the mixed layer increases the mixed layer potential temperature $\overline{\Theta }$ through detrainment (Fig. 5e).

The reason for this anomalous behavior is that this region is often covered with stratocumulus clouds^24–26^, characterized by low cloud cover that reflects sunlight. The marine stratocumulus is supported by the large-scale subsidence-induced capped-inversion^26–29^. Our composite shows that when ARs arrive, the SST is anomalously high (0.2–0.4 K), which weakens the capped-inversion. Meanwhile, the AR brings in a low-pressure system that creates ascending motion and further reduces subsidence. Therefore, the stratocumulus is much reduced (Fig. S5d, low cloud cover). On the other hand, the arrival of the AR increases mid and high cloud covers (Fig. S5b, c). Altogether, there is a net decrease in the total cloud cover, which in turn introduces sunlight (Fig. 5g). The shortwave radiation stratifies the surface ocean and shoals the MLD (Fig. 6e). In this case, the anomalous high SST produces anomalous cooling by increasing upward longwave radiation, which can be offset by the greenhouse effect due to high IWV^30^. Even more complicated, the high pre-existing SST is likely associated with ENSO, which increases the AR activity projected as EOF1 (also EOF2, but the mechanism is unclear). Our analysis aligns with Park and Leovy^31^, who found a negative correlation between the ENSO index and stratocumulus cloud frequency over the strong marine warming region. They argued that the reduction in stratiform cloud cover during ENSO is due to the increased SST, warm advection, and increased storm frequency.

One should not mistake strong marine warming events for marine heat waves. There are at least two differences between marine heat waves and strong marine warming events that we identify here. First, strong marine warming events refer to extreme SST tendencies, whereas marine heat waves are typically defined through extreme SST episodes. Second, marine heat waves require extreme SST to persist for over five days, which is much longer than the local lifetime of a typical AR event^32^.

## Discussion

The study of AR air–sea interaction remains under-explored. Shinoda et al.^14^ composited ARs by centering the IVT core to study ARs’ shared structure and common impact on the ocean. Their study underscored the importance of ocean entrainment in driving SST cooling, which is consistent with our findings. We have further found that the Ekman advection is another strong cooling response. Moreover, the SST tendency response map (Fig. 5) reveals several dynamically interesting regions. The first is coastal California, where advection causes anomalous warming rather than cooling. The second is the strong marine warming region, where the sensitivity of stratocumulus to the boundary and atmospheric conditions plays an important role. The third is the eddy-rich region along the Kuroshio Extension, where high variability shows the strong dependency of SST response to pre-existing conditions.

Given the strong SST modification due to ARs, the prediction skill of numerical models that do not use interactive ocean models (fixed-SST boundary condition) should dramatically decrease in time after each AR occurrence. This effect will be important in the case of multiple AR occurrences, known as atmospheric family events^33,34^, where later ARs interact with the SST anomalies induced by the earlier ARs. Moreover, an emerging consensus projects that ongoing global warming will lead to more frequent AR activity^35^, making AR impact on SST even more influential in the future. Our results suggest that the improvement of SST prediction during AR events using a full ocean model comes from resolving the change of SST due to surface fluxes, vertical mixing, and Ekman advection. In addition, the freshwater forcing associated with ARs might have a non-trivial role in air–sea interaction by modulating ocean stratification, and this topic requires more investigation.

Last but not least, frontal-scale SST structure is known to modify air–sea fluxes^36,37^. For example, SST eddies are shown to increase AR intensity^23^ by enhancing vertical moisture transport. However, ECCOv4 uses a coarse 1° resolution that is not eddy-resolving (although eddy effects are parameterized with a bolus velocity), and is a forced simulation that has no feedback to the atmosphere. Therefore, it is an open question as to what extent and how the co-evolving frontal-scale SST structure matters to ARs’ life cycle.

## Methods

### Datasets and AR condition

We obtain ocean potential temperature, salinity, and surface fluxes from Estimating the Circulation and Climate of the Ocean Version 4 release 4 (ECCOv4)^15,38^, which uses a 1° resolution MIT general circulation model (MITgcm)^39^ and reconstructs ocean states by assimilating observational data and forcing the ocean with adjusted ERA-interim air–sea fluxes. The ECCOv4 assimilation procedure does not nudge the model’s internal ocean state, meaning that the heat budget is exactly closed, and diagnostic terms needed to analyze this budget are made available by NASA Jet Propulsion Laboratory Physical Oceanography Distributed Active Archive Center (NASA JPL PODAAC). The ECCOv4 data extend from 1992 Jan 1 to 2018 Jan 1. This limits our target time interval to water years 1993–2017 (water year *n* starts Oct 1 of year *n*−1).

To compute IWV and IVT, we use the ECMWF Reanalysis–Interim (ERA-Interim)^16^, which has 60 vertical layers and a horizontal resolution of 79 km. We follow widely used definitions^12,40–42^ to compute these quantities as

2a $${{{{{{{\rm{IWV}}}}}}}}=-\frac{1}{g}\int\nolimits_{p={p}_{s}}^{200{{{{{{{\rm{hPa}}}}}}}}}q\,{{{{{{{\rm{d}}}}}}}}p,$$

2b $${{{{{{{\rm{IVT}}}}}}}}=\left| -\frac{1}{g}\int\nolimits_{p={p}_{s}}^{200{{{{{{{\rm{hPa}}}}}}}}}{{{{{{{\bf{v}}}}}}}}q\,{{{{{{{\rm{d}}}}}}}}p\right|,$$

where *q* is the specific humidity, *p*_*s*_ is the surface pressure, and **v** is the horizontal wind speed vector. To test the sensitivity, we have also used ECMWF Reanalysis v5 (ERA5)^43^, which has a horizontal resolution of 30 km and 137 vertical levels, to compute IWV and IVT. The outcome is almost identical to that of ERA-Interim. To detect ARs, we identify so-called AR objects. We adopt the definition from Guan and Waliser[^44^, hereafter GW15]. A valid AR object satisfies the following seven criteria: (1) Connected area where IVT is greater than a threshold. The threshold for a given day and location is defined as 100 kg/m/s or 85th percentile IVT of daily mean IVT within 15 days (i.e., previous 7 days, the given day, and post 7 days) using all 25 years, whichever is greater. (2) The length of the AR object, defined as the maximum great circle distance of the grids within the AR object, has to exceed 2000 km. This is a more selective criterion than GW15 used, but easier to define. (3) The overall orientation, defined as the great circle that measures the AR object length, cannot deviate more than 45° from the mean IVT vector of the AR object. (4) No more than 50% of the grid cells in the AR object can have IVT deviating 45° from the mean IVT. (5) The aspect ratio, defined as the square of the AR length divided by the area of the AR object, must be greater than 2. This is a stronger criterion than GW15 because the AR length we define is always greater than that defined by GW15. (6) AR objects cannot straddle the equator. (7) The mean poleward IVT of the AR object must be greater than 50 kg/m/s. Overall, our adopted AR detection algorithm is slightly more selective than GW15. We label a particular day for a given location as an AR day if an AR object covers the location.

In this study, we focus on the North Pacific $\left[1{0}^{\circ }\;{{{{{{{\rm{N}}}}}}}},6{0}^{\circ }\;{{{{{{{\rm{N}}}}}}}}\right]\times \left[12{0}^{\circ }\;{{{{{{{\rm{E}}}}}}}},12{0}^{\circ }\;{{{{{{{\rm{W}}}}}}}}\right]$. Since the ECCOv4 and ERA-Interim grids do not coincide, we regrid both datasets by taking the cosine-latitude-weighted-average of the data points that are within a common 2° × 2° box. Since we linearly regrid the data, and we compute the SST budget analysis using only the ECCOv4 data, regridding does not affect the closure of the SST budget.

### Mixed-layer diagnoses

The mixed layer is a well-mixed surface layer characterized by a low vertical potential density gradient. The MLD is defined as the shallowest depth at which the potential density exceeds the surface potential density by 0.03 kg m^−3^ ^45^. The potential density is computed using the nonlinear parameterization of Millero and Poisson^46^, which is consistent with ECCOv4^15,47^.

We briefly introduce how we obtain the SST budget equation for nonlinear free-surface formulation. In the MITgcm, the potential temperature tendency equation is^39,48^

3 $$\frac{\partial \Theta }{\partial t}=-\frac{1}{{\rho }_{0}}\frac{\partial F}{\partial z}-{\nabla }_{z}\cdot \left({{{{{{{\bf{v}}}}}}}}\Theta \right)-\frac{\partial w\Theta }{\partial z}+\frac{\partial }{\partial z}\left({K}_{V}\frac{\partial \Theta }{\partial z}\right)+{\nabla }_{z}\cdot \left({K}_{H}{\nabla }_{z}\Theta \right),$$

where vectors are denoted in bold, Θ is the ocean potential temperature, *ρ*_0_ is the reference seawater density, $\left({{{{{{{\bf{v}}}}}}}},w\right)=\left({{{{{{{{\bf{v}}}}}}}}}^{{{{{{{{\rm{Eu}}}}}}}}},{w}^{{{{{{{{\rm{Eu}}}}}}}}}\right)+\left({{{{{{{{\bf{v}}}}}}}}}^{{{{{{{{\rm{b}}}}}}}}},{w}^{{{{{{{{\rm{b}}}}}}}}}\right)$ is the residual ocean velocity as the sum of Eulerian and eddy-parametrizing bolus velocity, ∇_*z*_ is horizontal divergence (or gradient) operator with *z* held fixed, *K*_*V*_ and *K*_*H*_ are the vertical and horizontal diffusivity of potential temperature. In ECCOv4, *K*_*V*_ is determined using the Gaspar-Grégoris-Lefevre (GGL) turbulent kinetic energy parameterization scheme^49^. The surface flux *F* = *F*_sw_ + *F*_lw_ + *F*_sen_ + *F*_lat_ is the sum of shortwave radiation, longwave radiation, sensible heat flux, and latent heat flux (upward positive). The terms on the right-hand side of Equation (3) are external forcing, three-dimensional advective flux convergence, vertical diffusion, and horizontal diffusion.

Given an MLD, $h=h\left(x,y,t\right)$, and the surface height anomaly, $\eta=\eta \left(x,y,t\right)$, we define

4 $$\overline{\,\left(\,\cdot \,\right)\,}:=\frac{1}{h}\int\nolimits_{\eta -h}^{\eta }\,\left(\,\cdot \,\right)\,{{{{{{{\rm{d}}}}}}}}z$$

as the vertical averaging operator within the mixed layer. In the rest of the paper, we will use the mixed layer potential temperature $\overline{\Theta }$ as a proxy for SST.

Applying the averaging operator to (3), as shown in Supplementary Note 1, one may derive a mixed layer potential temperature tendency as

5 $${\dot{\overline{\Theta }}}_{{{{{{{{\rm{loc}}}}}}}}}={\dot{\overline{\Theta }}}_{{{{{{{{\rm{sw}}}}}}}}}+{\dot{\overline{\Theta }}}_{{{{{{{{\rm{lw}}}}}}}}}+{\dot{\overline{\Theta }}}_{{{{{{{{\rm{sen}}}}}}}}}+{\dot{\overline{\Theta }}}_{{{{{{{{\rm{lat}}}}}}}}}+{\dot{\overline{\Theta }}}_{{{{{{{{\rm{dilu}}}}}}}}}+{\dot{\overline{\Theta }}}_{{{{{{{{\rm{adv}}}}}}}}}+{\dot{\overline{\Theta }}}_{{{{{{{{\rm{vmix}}}}}}}}}+{\dot{\overline{\Theta }}}_{\det }+{\dot{\overline{\Theta }}}_{{{{{{{{\rm{hdif}}}}}}}}}.$$

where ${\dot{\overline{\Theta }}}_{{{{{{{{\rm{loc}}}}}}}}}=\partial \overline{\Theta }/\partial t$ is the local tendency of mixed layer potential temperature,

6 $${\dot{\overline{\Theta }}}_{{{{{{{{\rm{sw}}}}}}}}}=-\overline{\frac{1}{{\rho }_{0}}\frac{\partial {F}_{{{{{{{{\rm{sw}}}}}}}}}}{\partial z}}$$

7 $${\dot{\overline{\Theta }}}_{{{{{{{{\rm{lw}}}}}}}}}=-\overline{\frac{1}{{\rho }_{0}}\frac{\partial {F}_{{{{{{{{\rm{lw}}}}}}}}}}{\partial z}}$$

8 $${\dot{\overline{\Theta }}}_{{{{{{{{\rm{sen}}}}}}}}}=-\overline{\frac{1}{{\rho }_{0}}\frac{\partial {F}_{{{{{{{{\rm{sen}}}}}}}}}}{\partial z}}$$

9 $${\dot{\overline{\Theta }}}_{{{{{{{{\rm{lat}}}}}}}}}=-\overline{\frac{1}{{\rho }_{0}}\frac{\partial {F}_{{{{{{{{\rm{lat}}}}}}}}}}{\partial z}}$$

are the tendency due to the denoted surface heat fluxes, and

10 $${\dot{\overline{\Theta }}}_{{{{{{{{\rm{dilu}}}}}}}}}=\frac{{{{{{{{\rm{PmE}}}}}}}}}{{\rho }_{0}h}\left({\Theta }_{\eta }-\overline{\Theta }\right)$$

is the tendency due to the dilution effect of surface freshwater (PmE stands for precipitation minus evaporation mass flux in units of m/s, Θ_*η*_ is evaluated as the PmE potential temperature). The dilution term only tracks the flux of freshwater and does not account for the latent heat involved. In general, the dilution term is weak compared to other major contributions. The tendency of vertical mixing,

11 $${\dot{\overline{\Theta }}}_{{{{{{{{\rm{vmix}}}}}}}}}={\left({K}_{V}\frac{\partial \Theta }{\partial z}\right)}_{\eta -h}-\frac{\frac{\partial \left(h-\eta \right)}{\partial t}+\left| \frac{\partial \left(h-\eta \right)}{\partial t}\right| }{2h}\left(\overline{\Theta }-{\Theta }_{\eta -h}\right),$$

includes vertical diffusion and the tendency due to the deepening of the mixed layer. Although these two terms look different, they are meant to capture the vertical mixing due to entrainment (the turbulent vertical flux at the bottom of the mixed layer). The entrainment is captured in the first term for schemes parameterizing entrainment as enhanced *K*_*V*_, such as the GGL scheme^49^ used in ECCOv4 or the K-profile parameterization scheme^21,50^ used in many climate models. On the other hand, the entrainment is captured in the second term for schemes that directly solve for $\partial \left(h-\eta \right)/\partial t$, such as the Niiler-Kraus bulk formulation^22,51^. The tendency due to horizontal diffusion is

12 $${\dot{\overline{\Theta }}}_{{{{{{{{\rm{hdif}}}}}}}}}=\overline{{\nabla }_{z}\cdot \left({K}_{H}{\nabla }_{z}\Theta \right)}.$$

The tendency due to advection,

13 $${\dot{\overline{\Theta }}}_{{{{{{{{\rm{adv}}}}}}}}}=	-\overline{{{{{{{{\bf{v}}}}}}}}}\cdot {\nabla }_{z}\overline{\Theta }+\overline{{{{{{{{{\bf{v}}}}}}}}}^{{\prime} }\cdot {\nabla }_{z}{\Theta }^{{\prime} }}-\frac{{w}_{\eta -h}}{h}\left(\overline{\Theta }-{\Theta }_{\eta -h}\right) \\ 	-\overline{{{{{{{{\bf{v}}}}}}}}}\cdot \frac{{\nabla }_{z}\left(h-\eta \right)}{h}\left(\overline{\Theta }-{\Theta }_{\eta -h}\right)-\overline{{{{{{{{\bf{v}}}}}}}}}\cdot \frac{{\nabla }_{z}\eta }{h}\left({\Theta }_{\eta }-\overline{\Theta }\right),$$

includes the effects of mean flow advection, eddies (prime term ${\left(\cdot \right)}^{{\prime} }=\left(\cdot \right)-\overline{\left(\cdot \right)}$ is the departure from the vertical mean), and entrainment at the bottom of mixed layer due to residual velocities. The last term,

14 $${\dot{\overline{\Theta }}}_{\det }=\frac{\left| \frac{\partial \left(h-\eta \right)}{\partial t}\right| -\frac{\partial \left(h-\eta \right)}{\partial t}}{2h}\left(\overline{\Theta }-{\Theta }_{\eta -h}\right),$$

is the tendency due to detrainment, and it generally increases $\overline{\Theta }$ (because typically ∂Θ/∂*z* > 0). Notice that detrainment *does not change SST*. The awkward ${\dot{\overline{\Theta }}}_{\det }\ne 0$ is a construct due to the definition of vertical averaging. For convenience, we define

15 $${\dot{\overline{\Theta }}}_{{{{{{{{\rm{sfc}}}}}}}}}={\dot{\overline{\Theta }}}_{{{{{{{{\rm{sw}}}}}}}}}+{\dot{\overline{\Theta }}}_{{{{{{{{\rm{lw}}}}}}}}}+{\dot{\overline{\Theta }}}_{{{{{{{{\rm{sen}}}}}}}}}+{\dot{\overline{\Theta }}}_{{{{{{{{\rm{lat}}}}}}}}}+{\dot{\overline{\Theta }}}_{{{{{{{{\rm{dilu}}}}}}}}},$$

16 $${\dot{\overline{\Theta }}}_{{{{{{{{\rm{ocn}}}}}}}}}={\dot{\overline{\Theta }}}_{{{{{{{{\rm{adv}}}}}}}}}+{\dot{\overline{\Theta }}}_{{{{{{{{\rm{vmix}}}}}}}}}+{\dot{\overline{\Theta }}}_{\det }+{\dot{\overline{\Theta }}}_{{{{{{{{\rm{hdif}}}}}}}}},$$

as the overall tendency due to surface fluxes and ocean dynamics such that

17 $${\dot{\overline{\Theta }}}_{{{{{{{{\rm{loc}}}}}}}}}={\dot{\overline{\Theta }}}_{{{{{{{{\rm{sfc}}}}}}}}}+{\dot{\overline{\Theta }}}_{{{{{{{{\rm{ocn}}}}}}}}}.$$

The derivation of each tendency term from MITgcm is documented in Supplementary Note 2.

### Supplementary information

Supplementary Information

Peer Review File

## Data Availability

The data used to generate figures in this study have been deposited in Zenodo 10.5281/zenodo.10963326.

## Code availability

The code used to generate the figures in this study has been deposited in Zenodo 10.5281/zenodo.10958491 and Github https://github.com/meteorologytoday/paperfigures-2024-AR-SST-response/tree/v3.0.
