# Supplementary material for: Response of sea surface temperature to atmospheric rivers
Source: Nat Commun. 2024 Jun 12;15:5018. doi: 10.1038/s41467-024-48486-9 (PMC11169647; doi:10.1038/s41467-024-48486-9)
Supplement: Supplementary file 1 — Supplementary Information [file 41467_2024_48486_MOESM1_ESM.pdf]

Supplementary Information: Response of Sea Surface Temperature to Atmospheric Rivers

Tien-Yiao Hsu[1,*], Matthew R. Mazloff[1], Sarah T. Gille[1], Mara A. Freilich[2], Rui Sun[1], and Bruce D. Cornuelle[1]

[1]Scripps Institution of Oceanography, La Jolla, 92093, CA, United States
[2]Brown University, Department of Earth, Environmental and Planetary Sciences, Providence, 02912, RI, United States
[*]Corresponding author. Email: tienyiao@ucsd.edu

Supplementary Note 1: Derivation of Mixed Layer Potential Temperature tendency

According to Forget, G. et al. (2015)[1], the ECCO's on-transformed governing equation for potential temperature is

$$\frac{\partial\Theta}{\partial t}+\nabla_z\cdot(\Theta\mathbf{v})+\frac{\partial(\Theta w)}{\partial z}=\nabla_z\cdot\mathbf{F}_{\text{hdif}}+\frac{\partial F_{\text{vdif}}}{\partial z}-\frac{1}{\rho_0}\frac{\partial F_\Theta}{\partial z} \tag{1}$$

where vectors are denoted in bold, ∇_z is the horizontal divergence operator with fixed z, $(\mathbf{v},w)=\left(\mathbf{v}^{\text{Eu}},w^{\text{Eu}}\right)+\left(\mathbf{v}^b,w^b\right)$ is the residual velocity with $\left(\mathbf{v}^{\text{Eu}},w^{\text{Eu}}\right)$ being the Eulerian velocity and $\left(\mathbf{v}^b,w^b\right)$ the bolus velocity representing eddy mixing, $\mathbf{F}_{\text{hdif}}$ and F_{vdif} are the diffusive fluxes in horizontal and vertical directions, and $F_\Theta=F_{\text{sw}}+F_{\text{lw}}+F_{\text{sen}}+F_{\text{lat}}$ is the sum of upward fluxes including shortwave radiation, longwave radiation, sensible heat flux and latent heat flux. Let H be the ocean depth and $\eta=\eta(x,y,t)$ the sea surface height, z^*-coordinate is defined as $z^*=(z-\eta)/s^*$ where $s^*=1+\eta/H$ the geometrical factor. The transformed equation in the z^*-coordinate is[1]

$$\frac{\partial(s^*\Theta)}{\partial t}+\nabla_{z^*}\cdot(s^*\Theta\mathbf{v})+\frac{\partial(\Theta w)}{\partial z^*}=s^*\nabla_{z^*}\cdot\mathbf{F}_{\text{hdif}}+\frac{\partial F_{\text{vdif}}}{\partial z^*}-\frac{1}{\rho_0}\frac{\partial F_\Theta}{\partial z^*}. \tag{2}$$

The η evolves according to

$$\frac{\partial\eta}{\partial t}=\frac{\text{PmE}}{\rho_0}+w_\eta^{\text{Eu}} \tag{3}$$

where PmE is the precipitation and evaporation freshwater flux, and w_η^{Eu} is the Eulerian vertical velocity at the sea surface. We then define the mixed-layer average operator

$$\overline{(\cdot)}=\frac{1}{h^*}\int_{-h^*}^{0}(\cdot)\,\mathrm{d}z^*=\overline{(\cdot)}=\frac{1}{h}\int_{\eta-h}^{\eta}(\cdot)\,\mathrm{d}z. \tag{4}$$

The mixed-layer averaging comes with the following identities using fundamental theorem of calculus,

$$\frac{\partial}{\partial t}\overline{(\cdot)}=\overline{\frac{\partial(\cdot)}{\partial t}}-\frac{1}{h}\frac{\partial(h-\eta)}{\partial t}\left[\overline{(\cdot)}-(\cdot)_{z=\eta-h}\right]+\frac{1}{h}\frac{\partial\eta}{\partial t}\left[(\cdot)_{z=\eta}-\overline{(\cdot)}\right], \tag{5a}$$

$$\nabla_z\overline{(\cdot)}=\overline{\nabla_z(\cdot)}-\frac{1}{h}\nabla_z(h-\eta)\left[\overline{(\cdot)}-(\cdot)_{z=\eta-h}\right]+\frac{1}{h}\nabla_z\eta\left[(\cdot)_{z=\eta}-\overline{(\cdot)}\right]. \tag{5b}$$

Applying the mixed-layer averaging to (2), we derive

$$\frac{\partial\overline{\Theta}}{\partial t}=-\overline{\left(\mathbf{v}\cdot\nabla_z\Theta+w\frac{\partial\Theta}{\partial z}\right)}+\overline{\nabla_z\cdot\mathbf{F}_{\text{hdif}}}+\overline{\frac{\partial F_{\text{vdif}}}{\partial z}}+\frac{1}{\rho_0}\overline{\frac{\partial F_\Theta}{\partial z}}-\frac{1}{h}\frac{\partial(h-\eta)}{\partial t}\left(\overline{\Theta}-\Theta_{\eta-h}\right)+\frac{1}{h}\left(\frac{\text{PmE}}{\rho_0}+w_\eta^{\text{Eu}}\right)\left(\Theta_\eta-\overline{\Theta}\right), \tag{6}$$

where we substitute the tendency from (1) and use (3) to replace $\partial\eta/\partial t$. Using (5b) and letting $(\cdot)'=(\cdot)-\overline{(\cdot)}$, we can rewrite the advection term in z-coordinates as

$$\overline{\mathbf{v}\cdot\nabla_z\Theta+w\frac{\partial\Theta}{\partial z}}=\overline{\mathbf{v}}\cdot\nabla_z\overline{\Theta}+\overline{\mathbf{v}'\cdot\nabla_z\Theta'}+\overline{\mathbf{v}}\cdot\frac{\nabla_z(h-\eta)}{h}\left(\overline{\Theta}-\Theta_{\eta-h}\right)+\overline{\mathbf{v}}\cdot\frac{\nabla_z\eta}{h}\left(\Theta_\eta-\overline{\Theta}\right)$$
$$+\frac{w_\eta^{\text{Eu}}}{h}\left(\Theta_\eta-\overline{\Theta}\right)+\frac{w_{\eta-h}}{h}\left(\overline{\Theta}-\Theta_{\eta-h}\right) \tag{7}$$

where we also use the boundary condition $w_\eta^b = 0$ such that $w_\eta = w_\eta^{\mathrm{Eu}}$. We substitute (7) into (6) to obtain

$$\overbrace{\frac{\partial\overline{\Theta}}{\partial t}}^{\dot{\overline{\Theta}}_{\mathrm{loc}}} = \overbrace{\left(-\frac{1}{\rho_0}\frac{\partial F_{\mathrm{sw}}}{\partial z}\right)}^{\dot{\overline{\Theta}}_{\mathrm{sw}}} + \overbrace{\left(-\frac{1}{\rho_0}\frac{\partial F_{\mathrm{lw}}}{\partial z}\right)}^{\dot{\overline{\Theta}}_{\mathrm{lw}}} + \overbrace{\left(\frac{1}{\rho_0}\frac{\partial F_{\mathrm{sen}}}{\partial z}\right)}^{\dot{\overline{\Theta}}_{\mathrm{sen}}} + \overbrace{\left(-\frac{1}{\rho_0}\frac{\partial F_{\mathrm{lat}}}{\partial z}\right)}^{\dot{\overline{\Theta}}_{\mathrm{lat}}} + \overbrace{\frac{\mathrm{PmE}}{\rho_0 h}\left(\Theta_\eta - \overline{\Theta}\right)}^{\dot{\overline{\Theta}}_{\mathrm{dilu}}}$$
$$+ \overbrace{\left[-\overline{\mathbf{v}}\cdot\nabla_z\overline{\Theta} + \overline{\mathbf{v}'\cdot\nabla_z\Theta'} - \frac{w_{\eta-h}}{h}\left(\overline{\Theta} - \Theta_{\eta-h}\right) - \overline{\mathbf{v}}\cdot\frac{\nabla_z(h-\eta)}{h}\left(\overline{\Theta} - \Theta_{\eta-h}\right) - \overline{\mathbf{v}}\cdot\frac{\nabla_z\eta}{h}\left(\Theta_\eta - \overline{\Theta}\right)\right]}^{\dot{\overline{\Theta}}_{\mathrm{adv}}}$$
$$+ \overbrace{\left[\overline{\frac{\partial F_{\mathrm{vdif}}}{\partial z}} - \frac{\left|\frac{\partial(h-\eta)}{\partial t}\right| + \frac{\partial(h-\eta)}{\partial t}}{2h}\left(\overline{\Theta} - \Theta_{\eta-h}\right)\right]}^{\dot{\overline{\Theta}}_{\mathrm{vmix}}} + \overbrace{\left[\frac{\left|\frac{\partial(h-\eta)}{\partial t}\right| - \frac{\partial(h-\eta)}{\partial t}}{2h}\left(\overline{\Theta} - \Theta_{\eta-h}\right)\right]}^{\dot{\overline{\Theta}}_{\mathrm{det}}} + \overbrace{\overline{\nabla_z\cdot\mathbf{F}_{\mathrm{hdif}}}}^{\dot{\overline{\Theta}}_{\mathrm{hdif}}}. \tag{8}$$

Supplementary Note 2: Numerically Derive Terms in (8) using ECCO data

We can derive each grouped term in equation (8) as follows:

1. Determine mixed-layer depth (MLD) h.
2. Compute $\dot{\overline{\Theta}}_{\mathrm{loc}}$, $\dot{\overline{\Theta}}_{\mathrm{sw}}$, $\dot{\overline{\Theta}}_{\mathrm{lw}}$, $\dot{\overline{\Theta}}_{\mathrm{sen}}$, $\dot{\overline{\Theta}}_{\mathrm{lat}}$, $\dot{\overline{\Theta}}_{\mathrm{hdif}}$, $\dot{\overline{\Theta}}_{\mathrm{vmix}}$ and $(h-\eta)_t$. The computation of $\dot{\overline{\Theta}}_{\mathrm{loc}}$ and $(h-\eta)_t$ is exact because ECCO releases the snapshot output. The values of $\dot{\overline{\Theta}}_{\mathrm{hdif}}$and $\dot{\overline{\Theta}}_{\mathrm{hdif}}$ can also be computed exactly because ECCO releases the fluxes on grid faces. The fluxes at the bottom of the mixed layer is obtained by linearly interpolating the fluxes on the grid faces that encloses the desired depth.
3. Compute $\dot{\overline{\Theta}}_{\mathrm{dilu}}$ as explained in Section *Deriving Dilution Effect* $\dot{\overline{\Theta}}_{\mathrm{dilu}}$.
4. Compute $-\left(\overline{\Theta} - \Theta_{\eta-h}\right)\partial(h-\eta)/\partial t/h$ as explained in Section *Deriving the Bottom Boundary Flux.*
5. Now compute $\dot{\overline{\Theta}}_{\mathrm{vmix}}$ and $\dot{\overline{\Theta}}_{\mathrm{det}}$.
6. Compute $\dot{\overline{\Theta}}_{\mathrm{adv}}$ indirectly through equation (8).

Deriving the Bottom Boundary Flux

In this section, we introduce the method for obtaining the bottom boundary flux term in (8),

$$-\frac{\frac{\partial(h-\eta)}{\partial t}}{h}\left(\overline{\Theta} - \Theta_{\eta-h}\right), \tag{9}$$

which emerged in the identity (5a). For convenience, the variable $(\cdot)^n$ denotes the information at time $t_n = t_0 + n\Delta t$, i.e. $(\cdot)^n = (\cdot)(t = t_n = t_0 + n\Delta t)$. In the MITgcm, the potential temperature is stepped forward in time as[2]

$$\frac{\Theta^{n+1} - \Theta^n}{\Delta t} = G^{n+\frac{1}{2}}, \tag{10}$$

where Δt is the interval used to do time-stepping, and $G_{n+1/2}$ is the mean potential temperature tendency during the interval $t \in [t_n, t_{n+1}]$. We now write down the tendency of $\overline{\Theta}$ as

$$\frac{\overline{\Theta}^{n+1} - \overline{\Theta}^n}{\Delta t} = \frac{1}{h^{n+1}}\int_{\eta^{n+1}-h^{n+1}}^{\eta^{n+1}} \Theta^{n+1}\mathrm{d}z - \frac{1}{h^n}\int_{\eta^n-h^n}^{\eta^n} \Theta^n\mathrm{d}z. \tag{11}$$

We substitute Θ^{n+1} with (10), we can rearrange (11) into

$$\frac{\overline{\Theta}^{n+1} - \overline{\Theta}^n}{\Delta t} = \frac{1}{h^{n+1}}\int_{\eta^{n+1}-h^{n+1}}^{\eta^{n+1}} G^{n+\frac{1}{2}}\mathrm{d}z + \left(\frac{1}{h^{n+1}}\int_{\eta^{n+1}-h^{n+1}}^{\eta^{n+1}} \Theta^n\mathrm{d}z - \frac{1}{h^n}\int_{\eta^n-h^n}^{\eta^n} \Theta^n\mathrm{d}z\right). \tag{12}$$

The terms in the parenthesis can be rearranged as

$$\begin{aligned}
&\frac{1}{h^{n+1}}\int_{\eta^{n+1}-h^{n+1}}^{0}\Theta^n \mathrm{d}z+\frac{1}{h^{n+1}}\int_{0}^{\eta^{n+1}}\Theta^n \mathrm{d}z-\frac{1}{h^{n}}\int_{\eta^{n}-h^{n}}^{0}\Theta^n \mathrm{d}z-\frac{1}{h^{n}}\int_{0}^{\eta^{n}}\Theta^n \mathrm{d}z\\
=&\left(\frac{1}{h^{n+1}}\int_{\eta^{n+1}-h^{n+1}}^{0}\Theta^n \mathrm{d}z-\frac{1}{h^{n}}\int_{\eta^{n}-h^{n}}^{0}\Theta^n \mathrm{d}z\right)+\left(\frac{1}{h^{n+1}}\int_{0}^{\eta^{n+1}}\Theta^n \mathrm{d}z-\frac{1}{h^{n}}\int_{0}^{\eta^{n}}\Theta^n \mathrm{d}z\right),
\end{aligned} \tag{13}$$

where the physics reads that

$$-\frac{\frac{\partial(h-\eta)}{\partial t}}{h}\left(\overline{\Theta}-\Theta_{\eta-h}\right)\mapsto\frac{1}{h^{n+1}}\int_{\eta^{n+1}-h^{n+1}}^{0}\Theta^n \mathrm{d}z-\frac{1}{h^{n}}\int_{\eta^{n}-h^{n}}^{0}\Theta^n \mathrm{d}z. \tag{14}$$

This term is then counted as vertical mixing or detrainment according to the sign of $(h-\eta)_t$.

Deriving Dilution Effect $\dot{\overline{\Theta}}_{\mathrm{dilu}}$

Deriving the dilution effect $\dot{\overline{\Theta}}_{\mathrm{dilu}}$ needs two time-average products $\left\langle \mathrm{PmE}\left(\Theta_\eta-\overline{\Theta}\right)\right\rangle=\left\langle \mathrm{PmE}\,\Theta_\eta\right\rangle-\left\langle \mathrm{PmE}\,\overline{\Theta}\right\rangle$. The first term can be obtained explicitly with

$$\left\langle \mathrm{PmE}\,\Theta_\eta\right\rangle=\texttt{temp\_EvPrRn}\cdot\texttt{PmEpR}=\left(\texttt{TFLUX}-\texttt{oceQsw}-\texttt{EXFhl}-\texttt{EXFhs}+\texttt{EXFlwnet}\right)/c_p \tag{15}$$

whose detail derivation can be found in the Section *Derive* $\left\langle \mathrm{PmE}\,\Theta_\eta\right\rangle$ *in MITgcm*. For $\left\langle \mathrm{PmE}\,\overline{\Theta}\right\rangle$, we first expand each term with the decomposition $(\cdot)=\langle\cdot\rangle+(\cdot)^\star$ to see

$$\left\langle \mathrm{PmE}\,\overline{\Theta}\right\rangle=\langle\mathrm{PmE}\rangle\left\langle\overline{\Theta}\right\rangle+\left\langle \mathrm{PmE}^\star\,\overline{\Theta}^\star\right\rangle. \tag{16}$$

The second term on the right-hand-side is a time-correlation term. Since it is not obvious to us that higher (lower) $\overline{\Theta}$ implies higher (lower) PmE, we assume this term is negligible, meaning

$$\left\langle \mathrm{PmE}\left(\Theta_\eta-\overline{\Theta}\right)\right\rangle=\left\langle \mathrm{PmE}\,\Theta_\eta\right\rangle-\left\langle \mathrm{PmE}\,\overline{\Theta}\right\rangle=\left\langle \mathrm{PmE}\,\Theta_\eta\right\rangle-\langle\mathrm{PmE}\rangle\left\langle\overline{\Theta}\right\rangle-\cancel{\left\langle \mathrm{PmE}^\star\,\overline{\Theta}^\star\right\rangle}\approx\left\langle \mathrm{PmE}\,\Theta_\eta\right\rangle-\langle\mathrm{PmE}\rangle\left\langle\overline{\Theta}\right\rangle. \tag{17}$$

Therefore, we can compute

$$\dot{\overline{\Theta}}_{\mathrm{dilu}}=\frac{1}{\rho_0 h}\left\langle \mathrm{PmE}\left(\Theta_\eta-\overline{\Theta}\right)\right\rangle\approx\frac{1}{\rho_0 h}\left[\left\langle \mathrm{PmE}\,\Theta_\eta\right\rangle-\langle\mathrm{PmE}\rangle\left\langle\overline{\Theta}\right\rangle\right]. \tag{18}$$

Derive $\left\langle \mathrm{PmE}\,\Theta_\eta\right\rangle$ in MITgcm

The relations of heat budget variables in MITgcm[3] are listed below:

$$\begin{aligned}
\texttt{surForcT}&=\texttt{oceQnet}+\texttt{TRELAX}-\texttt{oceQsw}+c_p\cdot[\texttt{T_dilution_effect}], && \text{(19a)}\\
\texttt{surForcS}&=\texttt{oceSflux}+\texttt{SRELAX}+[\texttt{S_dilution_effect}], && \text{(19b)}\\
[\texttt{T_dilution_effect}]&=\texttt{PmEpR}\cdot(\texttt{temp_EvPrRn}-\texttt{SST}), && \text{(19c)}\\
[\texttt{S_dilution_effect}]&=\texttt{PmEpR}\cdot(\texttt{salt_EvPrRn}-\texttt{SSS}), && \text{(19d)}\\
\texttt{oceFWflx}&=[\texttt{PmEpR}], && \text{(19e)}\\
\texttt{TFLUX}&=\texttt{surForcT}+\texttt{oceQsw}+\texttt{oceFreez}+\texttt{PmEpR}\cdot\texttt{SST}\cdot c_p, && \text{(19f)}\\
\texttt{SFLUX}&=\texttt{surForcS}+\texttt{PmEpR}*\texttt{SSS}, && \text{(19g)}\\
\texttt{EXFqnet}&=\texttt{EXFlwnet}+\texttt{EXFswnet}-\texttt{EXFhl}-\texttt{EXFhs}, && \text{(19h)}
\end{aligned}$$

where list more equations than we need for future reference. For open-ocean, i.e., when there is no sea ice:

$$\begin{aligned}
\texttt{oceQnet}&=-\texttt{EXFqnet}, && \text{(20a)}\\
\texttt{oceQsw}&=-\texttt{EXFswnet}. && \text{(20b)}
\end{aligned}$$

We use (19a), (19c), (19f), (19h), (20a), and (20b) to derive

$$c_p\cdot\texttt{temp\_EvPrRn}\cdot\texttt{PmEpR}=\texttt{TFLUX}-\texttt{oceQsw}-\texttt{EXFhl}-\texttt{EXFhs}+\texttt{EXFlwnet} \tag{21}$$

Supplimentary Figures

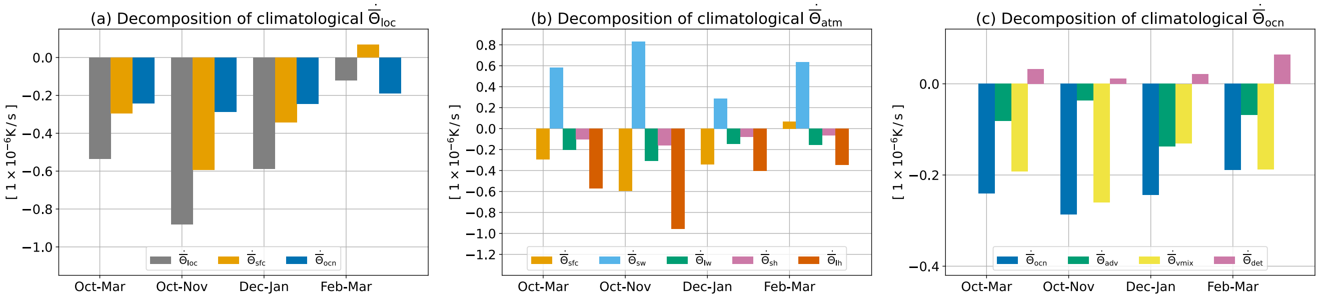

Figure S1. The sea surface temperature (SST) tendency analysis over atmospheric river active location $(35°N, 151°W)$. Each panel shows the breakdown of the climatology of SST tendency. (a) local SST tendency $\dot{\bar{\Theta}}_{loc}$, SST tendency due to surface fluxes $\dot{\bar{\Theta}}_{atm}$, and SST tendency due to ocean modification $\dot{\bar{\Theta}}_{ocn}$. (b) $\dot{\bar{\Theta}}_{atm}$ and its decomposition. (c) $\dot{\bar{\Theta}}_{ocn}$ and its decomposition.

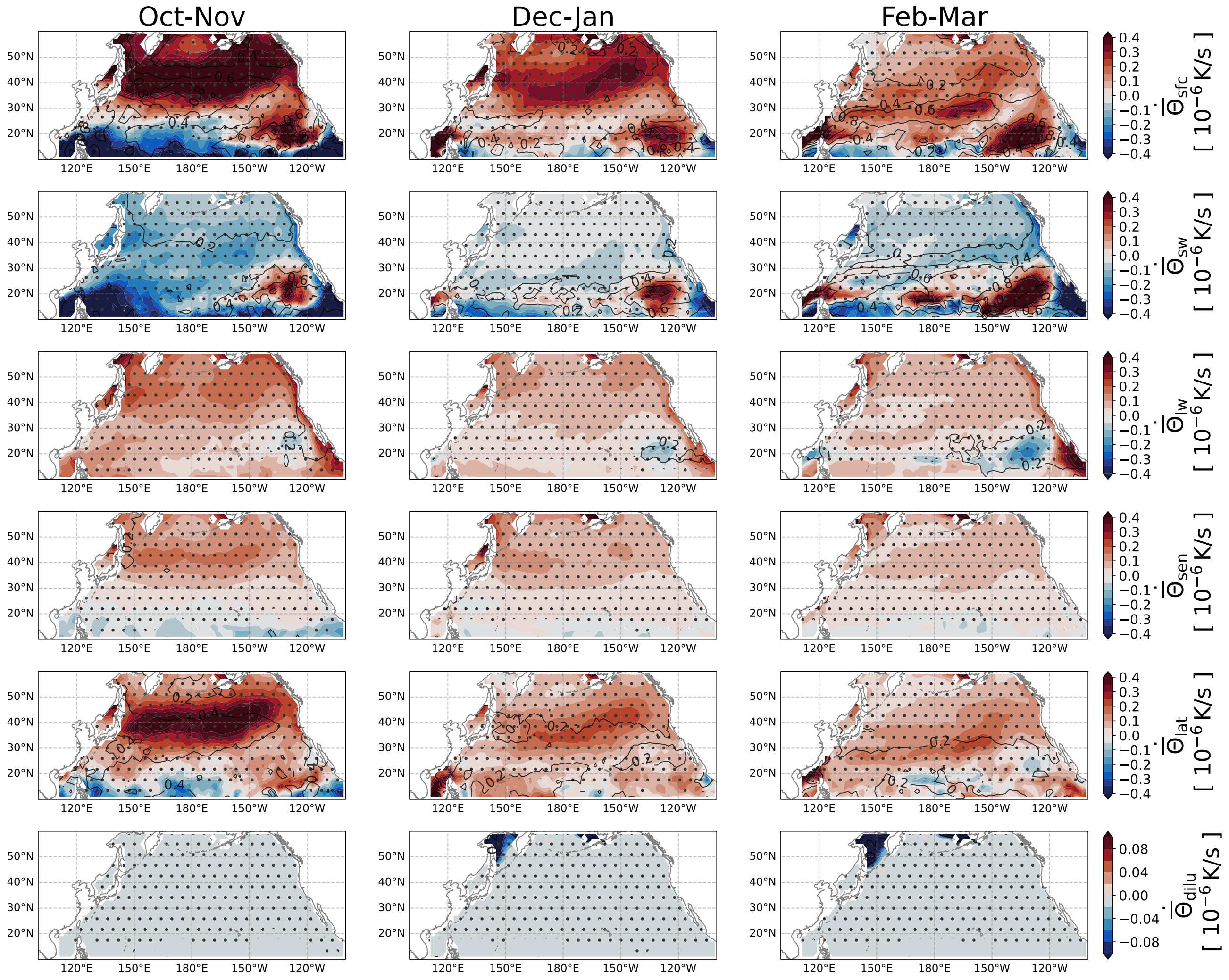

Figure S2. The decomposition of the response of sea surface temperature (SST) tendency to atmospheric rivers. Each panel shows the two-month mean (Oct–Nov, Dec–Jan, and Feb–Mar) of the composite SST tendency associated with a particular process (shading) and its standard deviation (contours). Each row shows anomalous SST tendency due to: (1) total surface fluxes $\dot{\overline{\Theta}}_{\mathrm{sfc}}$, (2) shortwave radiation $\dot{\overline{\Theta}}_{\mathrm{sw}}$, (3) longwave radiation $\dot{\overline{\Theta}}_{\mathrm{lw}}$, (4) sensible heat flux $\dot{\overline{\Theta}}_{\mathrm{sen}}$, (5) latent heat flux $\dot{\overline{\Theta}}_{\mathrm{lat}}$, and (6) dilution effect $\dot{\overline{\Theta}}_{\mathrm{dilu}}$. Dotted areas show response regions that pass the significance test ($p = 0.05$).

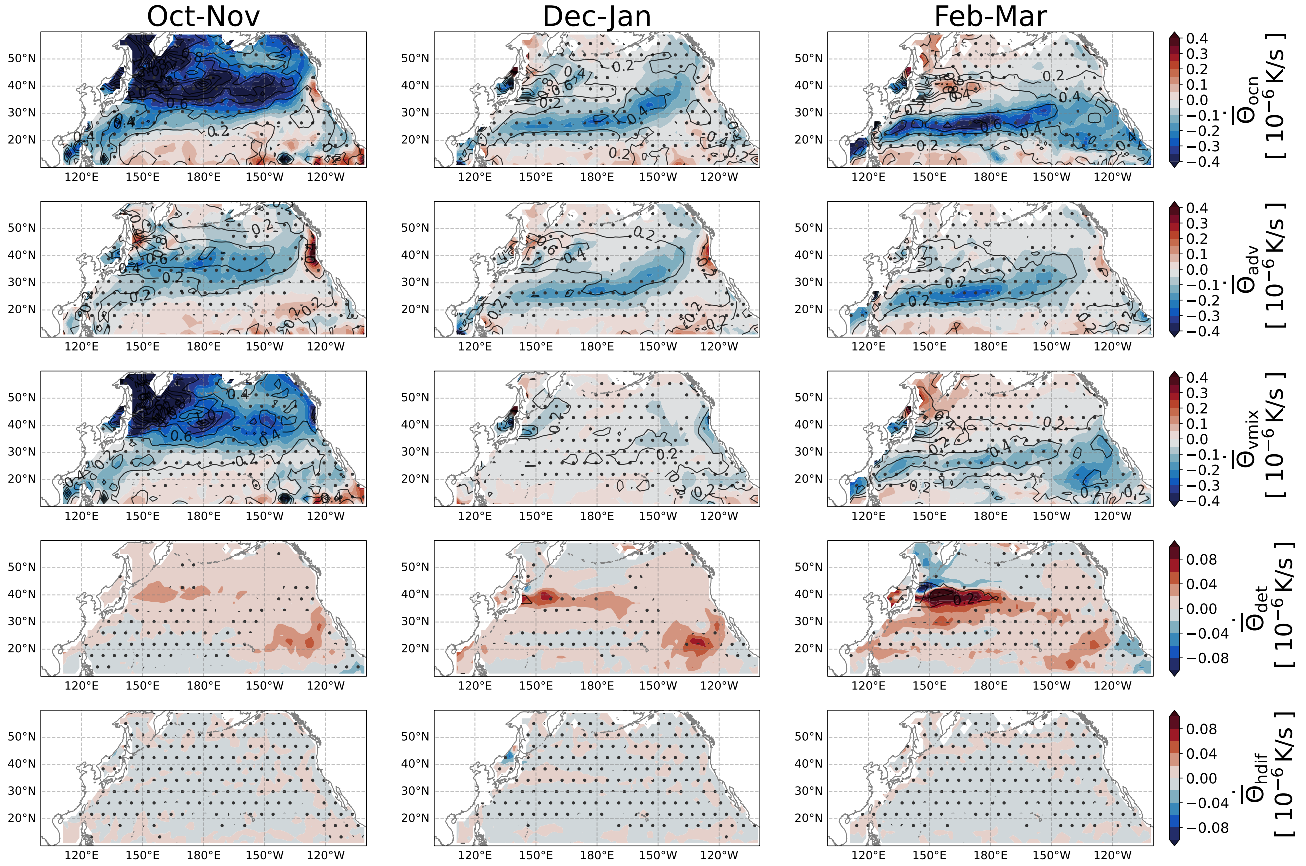

Figure S3. The decomposition of the response of sea surface temperature (SST) tendency to atmospheric rivers. Each panel shows the two-month mean (Oct–Nov, Dec–Jan, and Feb–Mar) of the composite SST tendency associated with a particular process (shading) and its standard deviation (contours). Each row shows anomalous SST tendency due to: (1) total ocean processes $\dot{\overline{\Theta}}_{\mathrm{ocn}}$, (2) advection $\dot{\overline{\Theta}}_{\mathrm{adv}}$, (3) vertical mixing $\dot{\overline{\Theta}}_{\mathrm{vmix}}$, (4) detrainment $\dot{\overline{\Theta}}_{\mathrm{det}}$, and (5) horizontal diffusion $\dot{\overline{\Theta}}_{\mathrm{hdif}}$. Dotted areas show response regions that pass the significance test ($p = 0.05$).

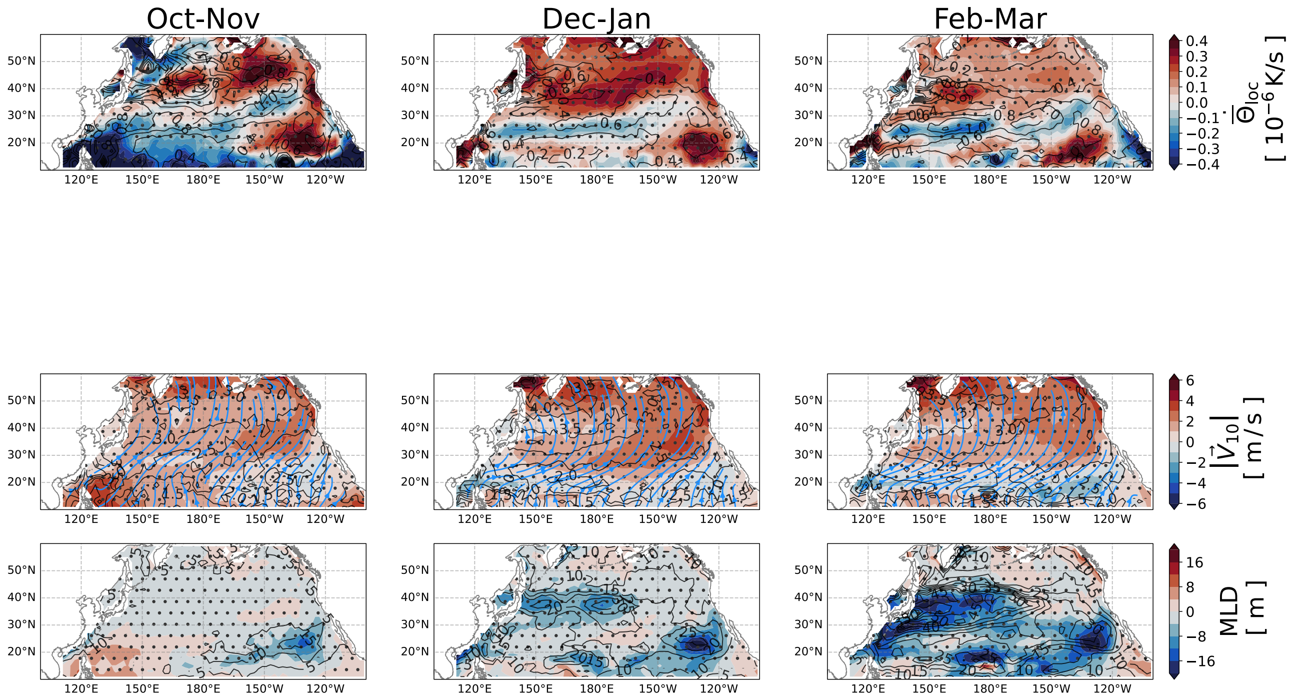

Figure S4. The decomposition of the response of sea surface temperature (SST) tendency to atmospheric rivers. Each panel shows the two-month mean (Oct–Nov, Dec–Jan, and Feb–Mar) of the composite SST tendency associated with a chosen variable (shading) and its standard deviation (contours). Each row shows (1) local anomalous SST tendency $\overline{\dot{\Theta}}_{\mathrm{loc}}$, (2) 10-m wind speed (shading) and direction (blue streamlines), and (3) mixed-layer depth (MLD). Dotted areas show response regions that pass the significance test ($p = 0.05$).

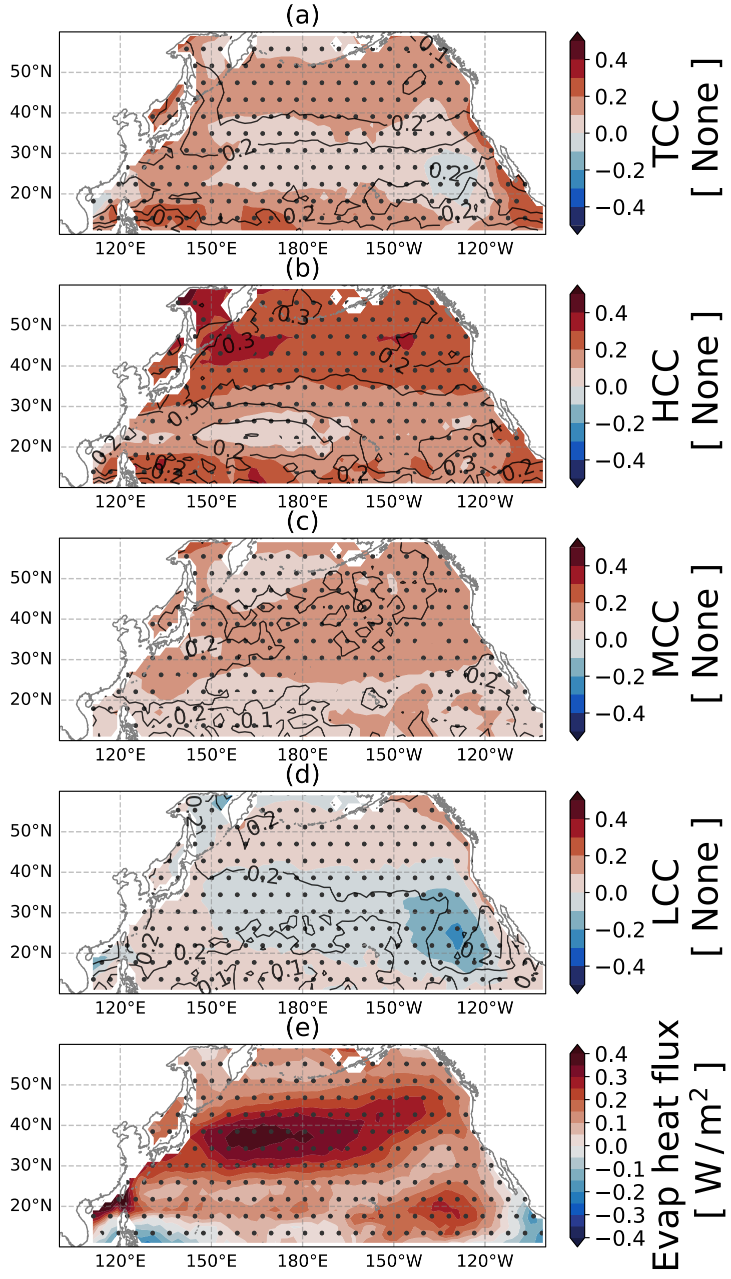

Figure S5. The composite of the mean (shading) and standard deviation (contours) of cloud response to atmospheric rivers during Oct–Mar. (a) total cloud cover, (b) high cloud cover, (c) mid cloud cover, and (d) low cloud cover. All the variables are measured in fractions, and therefore unitless. Dotted areas show response regions that pass the significance test ($p = 0.05$).

Supplementary References

1. Forget, G. *et al.* ECCO version 4: an integrated framework for non-linear inverse modeling and global ocean state estimation. *Geosci. Model. Dev. Discuss.* **8**, 3653–3743 (2015).
2. Piecuch, C. G. A note on practical evaluation of budgets in ECCO version 4 release 3. (2017).
3. Marshall, J., Adcroft, A., Hill, C., Perelman, L. & Heisey, C. A finite-volume, incompressible navier stokes model for studies of the ocean on parallel computers. *J. Geophys. Res.* **102**, 5753–5766 (1997).
