# Supplementary material for: Response of sea surface temperature to atmospheric rivers
Source: Nat Commun. 2024 Jun 12;15:5018. doi: 10.1038/s41467-024-48486-9 (PMC11169647; doi:10.1038/s41467-024-48486-9)
Supplement: Supplementary file 2 — Peer Review File [file 41467_2024_48486_MOESM2_ESM.pdf]

nature portfolio

Peer Review File

Response of Sea Surface Temperature to Atmospheric Rivers

Open Access This file is licensed under a Creative Commons Attribution 4.0 International License, which permits use, sharing, adaptation, distribution and reproduction in any medium or format, as long as you give appropriate credit to the original author(s) and the source, provide a link to the Creative Commons license, and indicate if changes were made. In the cases where the authors are anonymous, such as is the case for the reports of anonymous peer reviewers, author attribution should be to 'Anonymous Referee' followed by a clear attribution to the source work. The images or other third party material in this file are included in the article's Creative Commons license, unless indicated otherwise in a credit line to the material. If material is not included in the article's Creative Commons license and your intended use is not permitted by statutory regulation or exceeds the permitted use, you will need to obtain permission directly from the copyright holder. To view a copy of this license, visit http://creativecommons.org/licenses/by/4.0/.

REVIEWER COMMENTS

Reviewer #1 (Remarks to the Author):

Summary and major comments:

In "Response of Sea Surface Temperature to Atmospheric Rivers", the authors seek toward exploring the atmospheric river (AR)-induced impacts on sea surface temperatures (SSTs) over the North Pacific and their interactions with ocean dynamics. They identify some key oceanic processes under the AR influence that play the most important roles in modulating the SST tendency. These include ageostrophic advection, vertical diffusion, and entrainment.

The study focuses on the mixed-layer depth diagnoses based on a 25-year dataset and a decomposition of the local SST tendency. The authors present an interesting work in which they compare and explore various potential contributions of ARs to the development of SST anomalies. Their effort is timely and has potential to fill the gap in our understanding of AR feedback to oceanic forcing. However, there are some major issues that prevent me from suggesting an immediate publication. These include a) the AR definition used in this study is not appropriate, and b) the links between some oceanic processes and ARs are not well explained. My recommendation is that the manuscript be returned for a major revision.

Minor comments and suggestions:

Abstract: In the first sentence, ARs are responsible for SOME (not all) extreme weather conditions. In the sentence "Among all ocean processes, ageostrophic advection, vertical diffusion, and entrainment are the most important in modulating the SST tendency response", what are the contributions of ARs to these processes? In the sentence "For example …, sunlight", you could replace "marine warming region whose SST warming" with "marine region where SST warming"; how the disappearance of stratocumulus is related to AR activity? In the last sentence, consider replacing "the reduction" with "the AR-induced reduction".

L11: The two-week limit applies to the deterministic numerical weather prediction (NWP) methods thanks to the chaotic nature of the atmosphere. Some probabilistic forecast skill beyond this deterministic limit into the subseasonal-to-seasonal time scales (3 to 5 weeks) could be obtained by using ensemble NWP methods or through knowledge of some prominent air-sea oscillations, such as the Madden-Julian oscillation (Baggett et al. 2017; Lin et al. 2019).

L14-L16: Here, could you briefly mention the main mechanisms through which ARs insert their impacts on the oceans?

L28: Using daily climatology could include large day-to-day variability in your analysis. A 5-day or 10-day running climatology would be more appropriate.

L33: Consider replacing “two ridges” with “two variability ridges” or “two standard deviation ridges”.

L35-36: What is your definition of moderate correlation? The statistical significance of the correlation coefficient depends on the length of the data series N. |r| = 0.30 has different significance levels between N = 25 and N = 50. With only 25-year data, you should consider the AR correlation with ENSO, which is an interannual (5 to 7 years) oscillation, rather than with PDO, which is a decadal (or multi-decadal) oscillation. By the way, PDO has an ENSO-like pattern in SST.

L38-L40: EOF1 and EOF2 are supposed to be two independent modes. Do you use one physical mechanism to explain two independent modes?

L42: High IWV anomalies with ARs are not obvious in Fig. 1d.

L139: Could you explain briefly here why (how) AR conditions in this region would reduce the low cloud cover? And how about the AR-induced cloudiness?

L143-L148: Mo et al. (2022) investigated some AR-induced mechanisms for land heat waves, which could partly apply to the marine heat waves.

L149-L167: Could you include a remark about the implication of climate change on your findings? A warmer climate will certainly produce more and stronger ARs. Will it lead to larger SST anomalies?

L171: By “reduced shortwave radiation”, do you mean downward or upward?

L175: Is “incoastal” a typo?

L180-L195: You need to mention the period (1993-2017) of your study in this dataset description. You may also need to justify why this 25-year period is selected. Is it long enough to address decadal oscillation problem?

L185-L188: Atmospheric river can be generally defined as a long, narrow, and transient corridor of strong horizontal water vapor transport (https://glossary.ametsoc.org/wiki/Atmospheric_river). Specifically, most studies have a length requirement of 2000 km or at least 1500 km (Rutz et al. 2014; Guan & Waliser 2015; Mo et al. 2022). The AR definition used in this study is too simple. The minimum length of 500 km is too short, and without a requirement for an appropriate length/width ratio (e.g., > 2 in Guan & Waliser, 2015), some typhoons could be mis-identified as ARs. You need to either sharpen your AR definition or use one of the AR catalogues published by ARTMIP (Shields et al. 2018).

References:

Baggett, C. F., Barnes, E. A., Maloney, E. D., and Mundhenk, B. D., 2017: Advancing atmospheric river forecasts into subseasonal-to-seasonal time scales. Geophys. Res. Lett., 44, 7528–7536, https://doi.org/10.1002/2017GL074434.

Guan, B., and Waliser, D. E., 2015: Detection of atmospheric rivers: Evaluation and application of an algorithm for global studies. J. Geophys. Res. Atmos., 120, 12514–12535, https://doi.org/10.1002/2015JD024257.

Lin, H., Mo, R., Vitart, F., and Stan, C., 2019: Eastern Canada Flooding 2017 and its subseasonal predictions. Atmosphere-Ocean, 57, 195–207, https://doi.org/10.1080/07055900.2018.1547679.

Mo, R., Lin, H., and Vitart, F., 2022: An anomalous warm-season trans-Pacific atmospheric river linked to the 2021 western North America heatwave. Commun. Earth Environ., 3, 127, https://doi.org/10.1038/s43247-022-00459-w.

Rutz, J. J., Steenburgh, W. J., and Ralph, F. M., 2014: Climatological characteristics of atmospheric rivers and their inland penetration over the western United States. Mon. Wea. Rev., 142, 905–921, https://doi.org/10.1175/MWR-D-13-00168.1.

Shields, C. A., and Coauthors, 2018: Atmospheric River Tracking Method Intercomparison Project (ARTMIP): project goals and experimental design. Geosci. Model Dev., 11, 2455–2474, https://doi.org/10.5194/gmd-11-2455-2018.

Ruping Mo
Environment Climate Change Canada, Vancouver, BC, Canada
ruping.mo@ec.gc.ca

Reviewer #2 (Remarks to the Author):

Summary: In this study the authors use 25 years of ocean reanalysis data to discern how ARs affect SSTs over the North Pacific. They found that ARs have an overall warming effect on SSTs through reduced shortwave radiation and enhanced downward longwave radiation/sensible heat flux. This warming is offset up to 70% by ocean dynamics, namely ageostrophic advection and vertical mixing. The authors also showed that these SST responses to both surface and ocean forcing are not uniform throughout the North Pacific and also has some seasonal variability driven primarily by variations in the mixed layer depth as the cool season progresses. Shedding light on how ARs have a significant effect on SSTs and highlighting the need for interactive ocean models for subseasonal AR prediction is an invaluable contribution to the scientific community. Overall the paper is well written and structured and the author's assertions are supported by the figures and text, however, some further clarification is needed regarding the AR detection methodology used. In the opinion of this reviewer, this study adds to the existing body of scientific work in a tangible way and is worthy of publication with the revisions/comments outlined below.

Major Comments:

1. AR Detection Methodology - Please clarify what you mean by “interior points.” Additionally, are your results sensitive to AR length? 500km is very short, most AR studies require a standard length of at least 1500km for an anomalously high IVT/IWV region to be considered an AR.

Reference: Shields, Christine A., et al. "Atmospheric river tracking method intercomparison project (ARTMIP): project goals and experimental design." Geoscientific Model Development 11.6 (2018): 2455-2474.

Additionally, have you checked how AR detection frequency/location in ERA-Interim differs from AR detection frequency/location in ERA5?

2. Figure 4: How was the 10 day threshold chosen as a “valid year?” Are results sensitive to this threshold? I also don’t understand how only 5 of the 25 years used in this study had 10 AR days over a very active region throughout Feb-Mar.

3. Line 128: May be worth expanding on this a bit more. Yes, the anomalous local SST tendency does peak in Dec-Jan, but only because ocean damping is much weaker than the surface forcing. However, anomalous local SST tendencies forced by the surface and ocean components both peak in Oct-Nov. Because the ocean damping is much stronger in Oct-Nov than Dec-Jan it weakens the surface forcing more, giving the appearance of a peak in anomalous local SST tendency in Dec-Jan. The same conclusions can be drawn from Figure 4b and should be noted there as well around lines 92-93.

Minor Comments:

1. Instances where references are italicized should be changed to regular text (lines 18, 150, 199).
2. Figure 1: Need to denote what the dots in (d)-(f) indicate.
3. Lines 86-87: I don’t believe the argument can be made that there is warming due to dilution. Dilution actually looks mostly negligible or negative throughout Figure 4d and, by extension, 5g. 4. According to Figure 4 the warming is instead primarily due to increased sensible heating, longwave radiation and latent heating. Latent heating should be included in this sentence.
5. Figure 6: Panel d appears to only show the ageostrophic advection. Why is geostrophic advection mentioned in the caption? Should there be an additional panel showing geostrophic advection? If not please remove reference to geostrophic advection.
6. Line 115: Do you mean 6d instead of 6b?
7. Line 171: Still not sure reduced evaporation does much other than actually causing a cooling as shown by figures 4d and 5g. Would omit “reduced evaporation” from sentence.
8. In either the methods or results section please elaborate on why the study focuses only on Oct-Mar as opposed to the entire year.
9. Line 175: Add a space between “in” and “coastal”.
10. Equations 2a/b: Integral is backwards, p=ps should be on the bottom and 200 hPa should be on the top since it’s an atmospheric variable.
11. Lines 204 and 209: Please define GGL and KPP before using their acronyms.

12. Equations 6-9: Should there be negatives here? There are no negatives for these terms in equation 3 or supplemental information equation 8.
13. Supplemental Figures: Should note in the figure caption that it's the decomposition of the response of SST tendency to ARs broken down by 2 month periods.
14. Supplemental Figure 1: Note in figure caption that the blue contours indicate wind direction.
15. Supplemental Figure 3: Missing that row 4 is detrainment in figure caption, while horizontal diffusion should be noted as row 5.

Reviewer #3 (Remarks to the Author):

Review of "Response of Sea Surface Temperature to Atmospheric Rivers"

This manuscript analyzes the oceanic response to atmospheric rivers (AR) in the North Pacific region using an ocean reanalysis dataset. The main result is that ocean dynamics generally acts to damp the warming effect of ARs on SSTs north of 25N. Different terms in the mixed layer heat budget are analyzed to understand the oceanic response, which shows significant geographical variation.

The issue of oceanic response during AR events is important because the sea surface temperature (SST) response can act as a feedback that affects the subsequent atmospheric evolution of the weather system that drives the AR event. This manuscript makes a useful contribution to this topic and I would recommend publication after addressing the comments below.

Comments

Line 49: How well do the different terms in the SST budget balance? Is there a large residual term? Unlike in a model simulation, where all terms will balance, the reanalysis procedure may create spurious heat sources/sinks associated with adjustment of temperatures. Is this a significant effect?

Line 57-60: The slope of the relationship between Theta_ocn and Theta_sfc is quite small. Doesn't this mean that this explains only a small fraction of the overall variance?

Fig. 4: There is too much detail in this figure. It would be better to perhaps omit some of the information that is not relevant. Even for some other figures (e.g., Fig. 5), some the details are quite hard to read. The large version of the figures included at the end of the manuscript are helpful, but when the paper is printed, the small version of the figures may obscure the details.

Reply to Reviewer 1’s Comments on “Response of Sea Surface Temperature to Atmospheric Rivers”

Dear reviewer,

Thank you for your thorough review of our manuscript. Your feedback has been valuable in refining the content. In this revision, we have changed the dataset used to compute IVT from ERA5 to ERA-interim, and we also now use a widely accepted AR detection method. We will first elaborate on these changes, and then address each of your comments.

Use ERA-interim instead of ERA5 to compute IVT

In the original manuscript, we used ERA5 to compute IVT, while ECCOv4 uses ERA-interim to construct the reanalysis. Because this choice was questioned by some reviewers and is indeed a less ideal choice, in the revision, we recomputed IVT using ERA-interim data. We found little difference in IVT between these two datasets. Thus, although we have updated our reanalysis product, we have also concluded the IVT is not sensitive to the choice of datasets between ERA5 and ERA-interim.

An update on AR detection algorithm

Reviewers also raised concerns about our AR detection algorithm, specifically about the threshold of the size of ARs. In our original manuscript, we used 1000km as the length threshold (500km was a typo), which is smaller than the typical choice of 1500 to 2000km.

Given this concern from the reviewers, in this revision, we have updated the AR detection algorithm to approximately follow that of Guan & Waliser (2015) (GW15 afterwards):

1. IVT anomaly threshold is 85th monthly percentile IVT [15-day smoothed] or 100 kg m/s, whichever is greater.
2. The AR size (length) >= 2000km. Here, we define the length as the great arc of farthest points in the AR object. In GW15, they track the AR length on top of the great arc, meaning for the same given AR object, AR length defined in GW15 will always be greater than length defined by us. Therefore, we have a stricter criterion.
3. Aspect ratio length / width = length^2 / area > 2. Because our AR size is always equal or smaller than GW15’s AR length, for the same AR object, our algorithm gives an equal or smaller aspect ratio. This effectively makes this criterion stricter than GW15.

4. Direction check, meaning more than half of the grid points have an IVT vector that deviates less than 45 degrees from the mean IVT vector.
5. AR objects that straddle the equator are discarded, and
6. ARs whose poleward meridional IVT < 50 kg/m/s are discarded.

The change of AR detection results in fewer ARs detected in the middle of the Pacific but more along the coastline. Additionally, the revised AR detection removes ARs in low latitudes. EOF1 remains similar. The change in the second mode is that the new EOF2 has more emphasis over Japan and Eastern Pacific. This second mode better captures AR events that originate from the Eastern Pacific and influence the west coast America.

Old AR detection	New AR detection
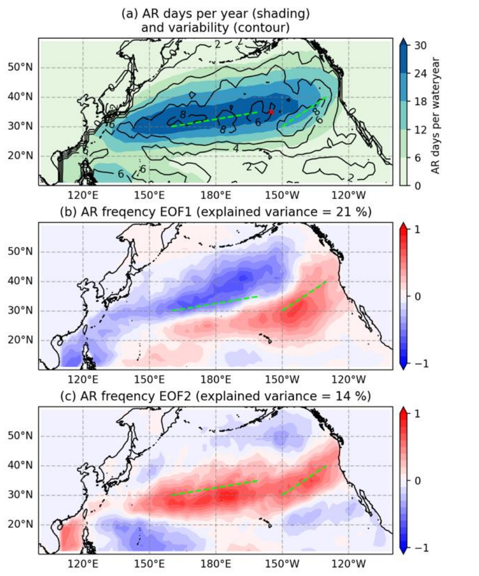	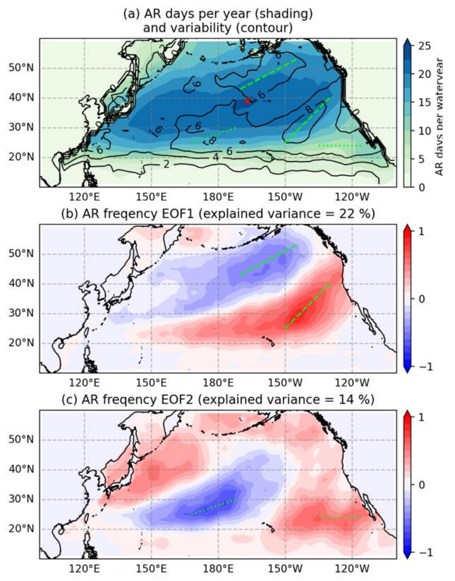

The change also affects the analysis in cases with negative surface forcing (AR cools the SST), as can be seen as follows

Old AR detection	New AR detection

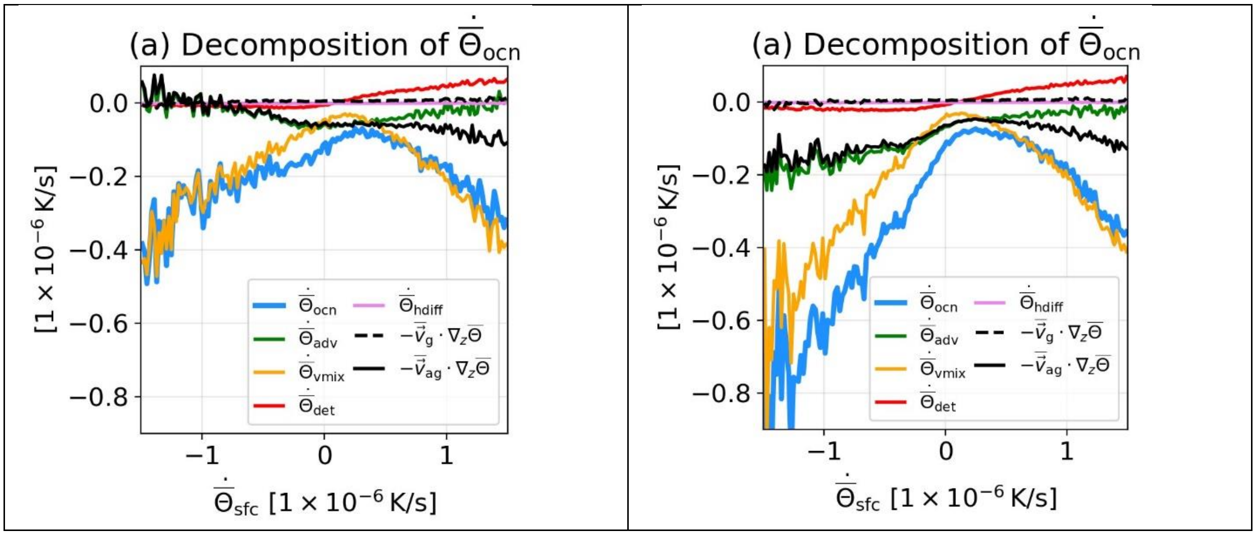

Reviewer #1 (Remarks to the Author):

Summary and major comments:

In “Response of Sea Surface Temperature to Atmospheric Rivers”, the authors seek toward exploring the atmospheric river (AR)-induced impacts on sea surface temperatures (SSTs) over the North Pacific and their interactions with ocean dynamics. They identify some key oceanic processes under the AR influence that play the most important roles in modulating the SST tendency. These include ageostrophic advection, vertical diffusion, and entrainment.

The study focuses on the mixed-layer depth diagnoses based on a 25-year dataset and a decomposition of the local SST tendency. The authors present an interesting work in which they compare and explore various potential contributions of ARs to the development of SST anomalies. Their effort is timely and has potential to fill the gap in our understanding of AR feedback to oceanic forcing. However, there are some major issues that prevent me from suggesting an immediate publication. These include a) the AR definition used in this study is not appropriate, and b) the links between some oceanic processes and ARs are not well explained. My recommendation is that the manuscript be returned for a major revision.

Reply: We address the AR-detection issue above.

Minor comments and suggestions:

Abstract: In the first sentence, ARs are responsible for SOME (not all) extreme weather conditions. In the sentence “Among all ocean processes, ageostrophic advection, vertical diffusion, and entrainment are the most important in modulating the SST tendency response”, what are the contributions of ARs to these processes? In the sentence “For example …, sunlight”, you could replace “marine warming region whose SST warming” with “marine region

where SST warming"; how the disappearance of stratocumulus is related to AR activity? In the last sentence, consider replacing "the reduction" with "the AR-induced reduction".

L11: The two-week limit applies to the deterministic numerical weather prediction (NWP) methods thanks to the chaotic nature of the atmosphere. Some probabilistic forecast skill beyond this deterministic limit into the subseasonal-to-seasonal time scales (3 to 5 weeks) could be obtained by using ensemble NWP methods or through knowledge of some prominent air-sea oscillations, such as the Madden-Julian oscillation (Baggett et al. 2017; Lin et al. 2019).

Reply:

Thank you for pointing this out. After some research, we found that the 10-14 day prediction skill seems to be documented by DeFlorio et al. (2018), who use "AR hits" as the metric. Incorporating this information, we have updated the text as follows:

"Past studies have identified predictors of AR activity, such as El Niño Southern Oscillation (ENSO) and Madden--Julian Oscillation [cite: Tseng et al. (2021), Lin et al. (2019), Baggett et al. (2017)]. However, skillful AR predictions do not exceed 10-20 days depending on the metric chosen [cite: DeFlorio et al. (2018), DeFlorio et al. (2019), Wick et al. (2013)]."

Reference:

DeFlorio, Michael J., et al. "Global assessment of atmospheric river prediction skill." Journal of Hydrometeorology 19.2 (2018): 409-426.

L14-L16: Here, could you briefly mention the main mechanisms through which ARs insert their impacts on the oceans?

Reply: We added the text to address this comment as follows: "Qualitatively, the physical expectation is that a relatively moist and warm AR heats the surface ocean through increasing downward heat fluxes. Simultaneously, its wind enhances vertical mixing at the base of the mixed layer that cools the surface ocean."

L28: Using daily climatology could include large day-to-day variability in your analysis. A 5-day or 10-day running climatology would be more appropriate.

Reply: As mentioned above, we changed the AR detection method. In this method, we obtain a monthly 85 percentile IVT field of 25 years using 15-day smoothing centered at each target date (i.e. 85 percentile of 25*15=375 samplings). This process removes large day-to-day variability.

L33: Consider replacing "two ridges" with "two variability ridges" or "two standard deviation ridges".

Reply: We have revised the language. Also, after updating the AR detection algorithm we now find four variability ridges, and these spatially match the locations of bigger amplitudes in EOF1 and EOF2.

L35-36: What is your definition of moderate correlation? The statistical significance of the correlation coefficient depends on the length of the data series N. |r| = 0.30 has different significance levels between N = 25 and N = 50. With only 25-year data, you should consider the AR correlation with ENSO, which is an interannual (5 to 7 years) oscillation, rather than with PDO, which is a decadal (or multi-decadal) oscillation. By the way, PDO has an ENSO-like pattern in SST.

See the next comment.

L38-L40: EOF1 and EOF2 are supposed to be two independent modes. Do you use one physical mechanism to explain two independent modes?

Reply: No, our intent was only to make connections between ENSO and EOF1 & EOF2. And we should not use one mechanism to explain both EOFs since they are temporally independent. We have rewritten the text in L37-45 as

“EOF1 and EOF2 roughly represent the shift of AR activity south to north and west to east, respectively. EOF1 positively correlates with the NINO3.4 SST index (95% confidence interval). This connection arises because ENSO events tend to deepen the Aleutian Low, which controls the location of stormtrack activity [citation]. This result is consistent with Tseng et al. (2021) who examined this relationship more closely. EOF2 positively correlates with NINO3.4 SST index (80% confidence interval) as well, although the correlation is weaker. These results indicate that ENSO can change ARs' north-south and east-west frequency. A deeper physical understanding is beyond the scope of this paper and will require future investigations.”

L42: High IWV anomalies with ARs are not obvious in Fig. 1d.

Reply: We were showing the composited total IWV. Now we have changed it to show composited anomalous IWV.

Original Manuscript

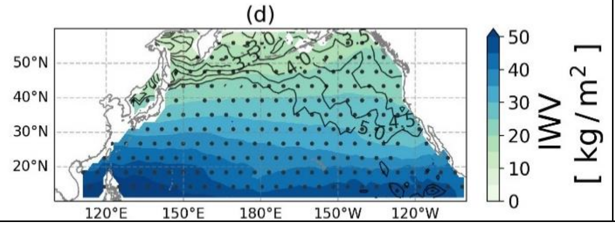

Revision

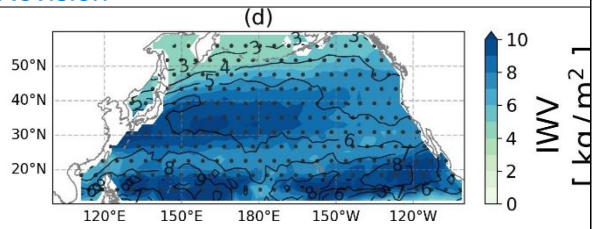

L139: Could you explain briefly here why (how) AR conditions in this region would reduce the low cloud cover? And how about the AR-induced cloudiness?

Reply:

We think that ARs are typically in the vicinity of low-pressure systems that bring in large-scale convergence. The large-scale convergence removes the capping inversion, which does not favor the formation of marine stratocumulus (Lily 1968, Kuo and Schubert 1988). Also, the composited SST shows that the SST is on average 0.2K~0.4K warmer than climatology. This is an indication of ENSO. Therefore, the reduction of subsidence can also be partially due to large-scale warm SST. The reduced subsidence does not favor marine stratocumulus, and thus it is reduced. The mid and high clouds brought in by ARs cannot fully compensate the loss of low cloud, and there is a net reduction of total cloud cover.

However, the story is complicated by ENSO increasing AR frequency over the eastern Pacific. It will take a separate study to explore air-sea interaction in this region.

We have rewritten the text in L158-169.

L143-L148: Mo et al. (2022) investigated some AR-induced mechanisms for land heat waves, which could partly apply to the marine heat waves.

Reply: We have added some text that mentions the possible greenhouse effect due to high IWV, which might reduce the longwave cooling.

L149-L167: Could you include a remark about the implication of climate change on your findings? A warmer climate will certainly produce more and stronger ARs. Will it lead to larger SST anomalies?

Reply: We added the text as follows (bold font below; in L184-186 of the paper)

"…This effect will be important in the case of multiple AR occurrences, known as atmospheric family events [cite Fish et al. (2022), Fish et al. (2019)] **where the later ARs will interact with the SST anomalies induced by the earlier ARs**. **Moreover, an emerging consensus projects that ongoing global warming will lead to more frequent AR activity [cite Payne et al. 2020], making AR impact on SST even more influential in the future.**"

L171: By "reduced shortwave radiation", do you mean downward or upward?

Reply: We mean downward. In this revision, we have added "downward" to make it clearer.

L175: Is "incoastal" a typo?

Reply: Yes it is a typo. We corrected it to "... response in coastal California…"

L180-L195: You need to mention the period (1993-2017) of your study in this dataset description. You may also need to justify why this 25-year period is selected. Is it long enough to address decadal oscillation problem?

Reply: The ECCOv4 data spans from 1992 Jan 1 to 2018 Jan 1. This limits our target time interval down to water years 1993-2017 (water year n starts Oct 1 of year $n - 1$). We have added this information to the Method section.

Also, we agree that it is a stretch to relate a 25-year record to the PDO, and we now focus on their relationship to ENSO as explained in the previous comments.

L185-L188: Atmospheric river can be generally defined as a long, narrow, and transient corridor of strong horizontal water vapor transport (https://glossary.ametsoc.org/wiki/Atmospheric_river). Specifically, most studies have a length requirement of 2000 km or at least 1500 km (Rutz et al. 2014; Guan & Waliser 2015; Mo et al. 2022). The AR definition used in this study is too simple. The minimum length of 500 km is too short, and without a requirement for an appropriate length/width ratio (e.g., > 2 in Guan & Waliser, 2015), some typhoons could be mis-identified as ARs. You need to either sharpen your AR definition or use one of the AR catalogues published by ARTMIP (Shields et al. 2018).

Reply: We have updated the AR detection method as explained in the beginning of this document.

References:

Baggett, C. F., Barnes, E. A., Maloney, E. D., and Mundhenk, B. D., 2017: Advancing atmospheric river forecasts into subseasonal-to-seasonal time scales. Geophys. Res. Lett., 44, 7528–7536, https://doi.org/10.1002/2017GL074434.

Guan, B., and Waliser, D. E., 2015: Detection of atmospheric rivers: Evaluation and application of an algorithm for global studies. J. Geophys. Res. Atmos., 120, 12514–12535, https://doi.org/10.1002/2015JD024257.

Lin, H., Mo, R., Vitart, F., and Stan, C., 2019: Eastern Canada Flooding 2017 and its subseasonal predictions. Atmosphere-Ocean, 57, 195–207, https://doi.org/10.1080/07055900.2018.1547679.

Mo, R., Lin, H., and Vitart, F., 2022: An anomalous warm-season trans-Pacific atmospheric river linked to the 2021 western North America heatwave. Commun. Earth Environ., 3, 127, https://doi.org/10.1038/s43247-022-00459-w.

Rutz, J. J., Steenburgh, W. J., and Ralph, F. M., 2014: Climatological characteristics of atmospheric rivers and their inland penetration over the western United States. Mon. Wea. Rev., 142, 905–921, https://doi.org/10.1175/MWR-D-13-00168.1.

Shields, C. A., and Coauthors, 2018: Atmospheric River Tracking Method Intercomparison Project (ARTMIP): project goals and experimental design. Geosci. Model Dev., 11, 2455–2474, https://doi.org/10.5194/gmd-11-2455-2018.

Reviewer #2 (Remarks to the Author):

Summary: In this study the authors use 25 years of ocean reanalysis data to discern how ARs affect SSTs over the North Pacific. They found that ARs have an overall warming effect on SSTs through reduced shortwave radiation and enhanced downward longwave radiation/sensible heat flux. This warming is offset up to 70% by ocean dynamics, namely ageostrophic advection and vertical mixing. The authors also showed that these SST responses to both surface and ocean forcing are not uniform throughout the North Pacific and also has some seasonal variability driven primarily by variations in the mixed layer depth as the cool season progresses. Shedding light on how ARs have a significant effect on SSTs and highlighting the need for interactive ocean models for subseasonal AR prediction is an invaluable contribution to the scientific community. Overall the paper is well written and structured and the author’s assertions are supported by the figures and text, however, some further clarification is needed regarding the AR detection methodology used. In the opinion of this reviewer, this study adds to the existing body of scientific work in a tangible way and is worthy of publication with the revisions/comments outlined below.

Major Comments:

1. AR Detection Methodology - Please clarify what you mean by “interior points.” Additionally, are your results sensitive to AR length? 500km is very short, most AR studies require a standard length of at least 1500km for an anomalously high IVT/IWV region to be considered an AR.

Reference: Shields, Christine A., et al. "Atmospheric river tracking method intercomparison project (ARTMIP): project goals and experimental design." Geoscientific Model Development 11.6 (2018): 2455-2474.

Additionally, have you checked how AR detection frequency/location in ERA-Interim differs from AR detection frequency/location in ERA5?

Reply: We have addressed this comment above.

2. Figure 4: How was the 10 day threshold chosen as a “valid year?” Are results sensitive to this threshold? I also don’t understand how only 5 of the 25 years used in this study had 10 AR days over a very active region throughout Feb-Mar.

Reply:

Because we changed the AR detection algorithm, which shifted the average AR position, we have changed the point location for the analysis in Figure 4. We picked the new location at [35N, 155W] where the ocean’s modification can be better demonstrated. As for the threshold sensitivity, our tests show that there is an insignificant difference between 3, 5 and 10 AR-days, while 15 AR-days greatly reduces the sampling size, such that the outcome is meaningless. In this revision, we picked 5 days to include most of the wateryears, and we have updated the text to address the sensitivity. Finally, we show these sensitivity tests here for your reference.

Threshold = 3 AR-days

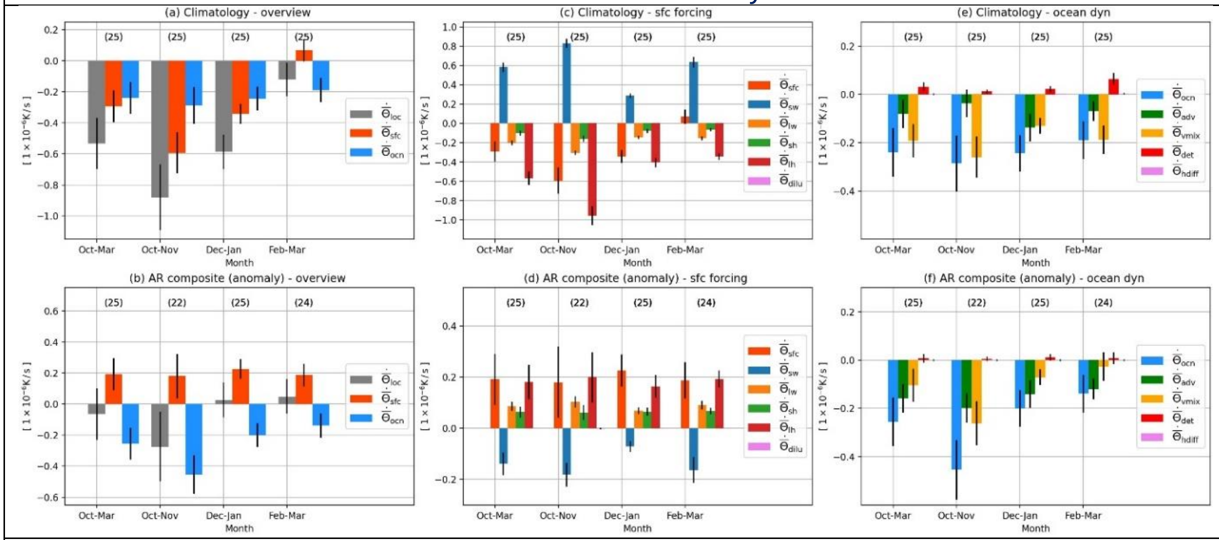

Threshold = 5 AR-days

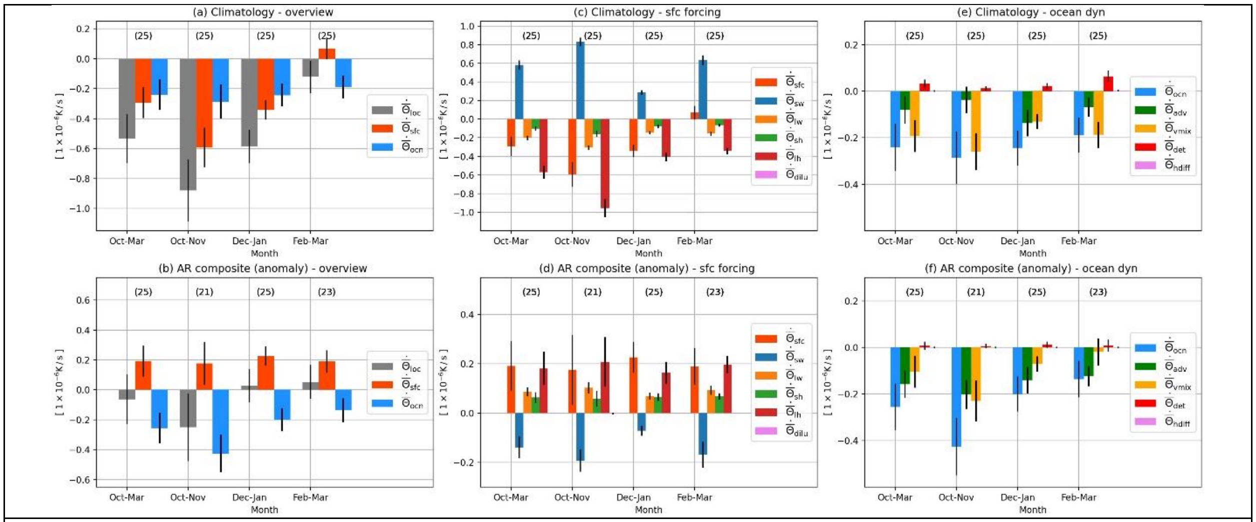

Threshold = 10 AR-days

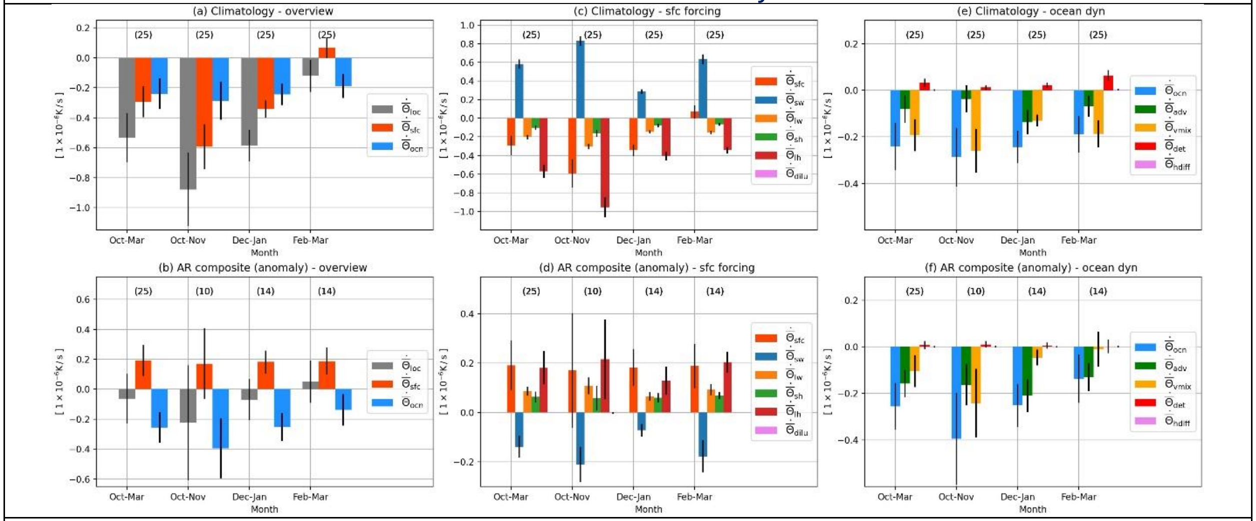

Threshold = 15 AR-days

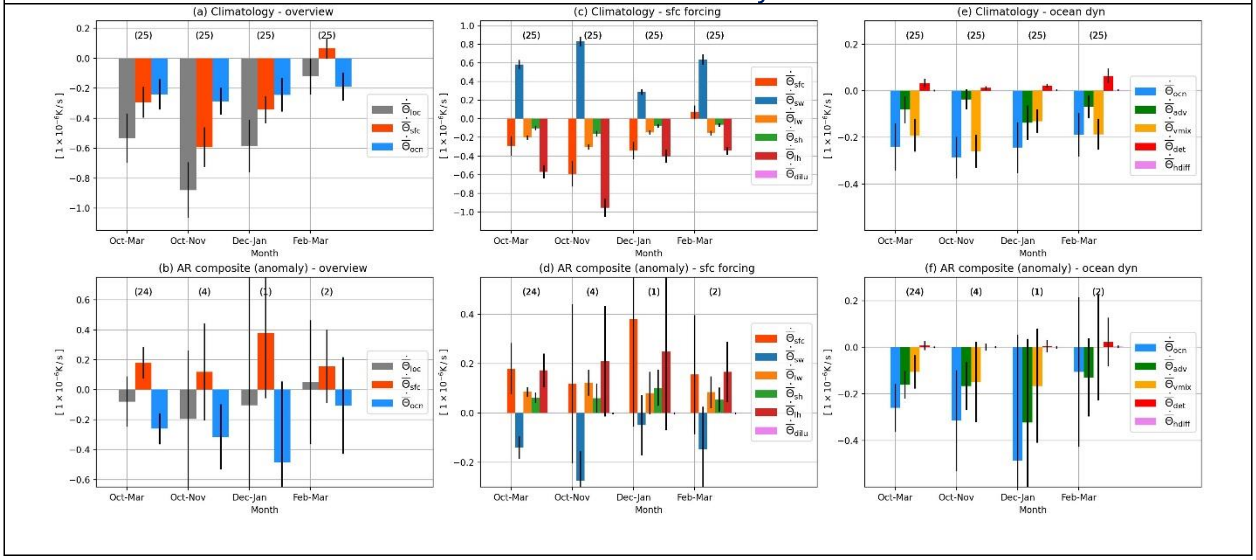

3. Line 128: May be worth expanding on this a bit more. Yes, the anomalous local SST tendency does peak in Dec-Jan, but only because ocean damping is much weaker than the surface forcing. However, anomalous local SST tendencies forced by the surface and ocean components both peak in Oct-Nov. Because the ocean damping is much stronger in Oct-Nov than Dec-Jan it weakens the surface forcing more, giving the appearance of a peak in anomalous local SST tendency in Dec-Jan. The same conclusions can be drawn from Figure 4b and should be noted there as well around lines 92-93.

Reply: We have rewritten and expanded the discussion on seasonality.

Minor Comments:

1. Instances where references are italicized should be changed to regular text (lines 18, 150, 199).

Reply: Updated as suggested.

2. Figure 1: Need to denote what the dots in (d)-(f) indicate.

Reply: We have added this information.

3. Lines 86-87: I don't believe the argument can be made that there is warming due to dilution. Dilution actually looks mostly negligible or negative throughout Figure 4d and, by extension, 5g.

4. According to Figure 4 the warming is instead primarily due to increased sensible heating, longwave radiation and latent heating. Latent heating should be included in this sentence.

Reply to 3 and 4: We do not argue that dilution plays an important role in SST warming. We only present the dilution effect in the figure for completeness.

5. Figure 6: Panel d appears to only show the ageostrophic advection. Why is geostrophic advection mentioned in the caption? Should there be an additional panel showing geostrophic advection? If not please remove reference to geostrophic advection.

Reply: This is a typo. Geostrophic response is too weak to be shown. We have fixed the caption.

6. Line 115: Do you mean 6d instead of 6b?

Reply: This is a typo. We have fixed the text.

7. Line 171: Still not sure reduced evaporation does much other than actually causing a cooling as shown by figures 4d and 5g. Would omit "reduced evaporation" from sentence.

Reply: The titles of the subfigures might have confused the reviewers. In the previous version of the manuscript he 5g was a dilution effect, which is negligible. (We have dropped it in the revision.) The new Figure 5j shows the strong anomalous warming due to latent heating. Because latent heating only involves evaporation, the explanation can only be reduced evaporation.

8. In either the methods or results section please elaborate on why the study focuses only on Oct-Mar as opposed to the entire year.

Reply: We add in the text at the beginning of result section: “In this paper, we focus on Oct--Mar when ARs are the most active in the North Pacific.”

9. Line 175: Add a space between “in” and “coastal”.

Reply: We have fixed the text as suggested.

10. Equations 2a/b: Integral is backwards, p=ps should be on the bottom and 200 hPa should be on the top since it’s an atmospheric variable.

Reply: We change the integral bounds as follows.

$$\mathrm{IWV} = -\frac{1}{g}\int_{p=p_s}^{200\mathrm{hPa}} q\ \mathrm{d}p$$

and

$$\mathrm{IVT} = \left| -\frac{1}{g}\int_{p=p_s}^{200\mathrm{hPa}} q\vec{V}\ \mathrm{d}p \right|$$

Which is the same mathematical form as ARTMIP whitepaper (Christian et al. 2018).

11. Lines 204 and 209: Please define GGL and KPP before using their acronyms.

Reply: Updated as suggested.

12. Equations 6-9: Should there be negatives here? There are no negatives for these terms in equation 3 or supplemental information equation 8.

Reply: Yes. We have fixed the equation and noted these fluxes are upward positive.

13. Supplemental Figures: Should note in the figure caption that it’s the decomposition of the response of SST tendency to ARs broken down by 2 month periods.

Reply: We have added this information.

14. Supplemental Figure 1: Note in figure caption that the blue contours indicate wind direction.

Reply: We have added this information.

15. Supplemental Figure 3: Missing that row 4 is detrainment in figure caption, while horizontal diffusion should be noted as row 5.

Reply: We have added this information.

Reviewer #3 (Remarks to the Author):

Review of “Response of Sea Surface Temperature to Atmospheric Rivers”

This manuscript analyzes the oceanic response to atmospheric rivers (AR) in the North Pacific region using an ocean reanalysis dataset. The main result is that ocean dynamics generally acts to damp the warming effect of ARs on SSTs north of 25N. Different terms in the mixed layer heat budget are analyzed to understand the oceanic response, which shows significant geographical variation.

The issue of oceanic response during AR events is important because the sea surface temperature (SST) response can act as a feedback that affects the subsequent atmospheric evolution of the weather system that drives the AR event. This manuscript makes a useful contribution to this topic and I would recommend publication after addressing the comments below.

Comments

Line 49: How well do the different terms in the SST budget balance? Is there a large residual term? Unlike in a model simulation, where all terms will balance, the reanalysis procedure may create spurious heat sources/sinks associated with adjustment of temperatures. Is this a significant effect?

Reply: There is no residual. Compared to other reanalyses, ECCOv4 is unique in that the 4D-variational data assimilation occurs over a single assimilation period. The 1992 initial conditions are adjusted, as are the atmospheric state and mixing parameters, but there is no nudging throughout the ~30-year window. All terms to close the budget to machine precision are available via PO.DAAC (https://podaac.jpl.nasa.gov/ECCO). We have noted this in the text by adding the sentence, “The ECCOv4 assimilation procedure does not nudge the model internal ocean state, meaning that the heat budget is exactly closed, and all budget terms needed to close this budget are available from the NASA Jet Propulsion Laboratory Physical Oceanography Distributed Active Archive Center (NASA JPL PODAAC).”

Finally, to avoid confusion, we have changed the abbreviation “ECCO” to “ECCOv4” in this paper.

Line 57-60: The slope of the relationship between Theta_ocn and Theta_sfc is quite small. Doesn’t this mean that this explains only a small fraction of the overall variance?

Reply: Our intent is to show that their relationship is nonlinear by computing a linear fit and showing its low R value. We additionally include a bin-averaged dotted blue line to roughly show a non-linear relationship with two regimes split by $\overline{\dot{\Theta}}_{\mathrm{sfc}} = 0$.

Fig. 4: There is too much detail in this figure. It would be better to perhaps omit some of the information that is not relevant. Even for some other figures (e.g., Fig. 5), some the details are quite hard to read. The large version of the figures included at the end of the manuscript are helpful, but when the paper is printed, the small version of the figures may obscure the details.

Reply: In this revision, we split the figure by rows: climatology (upper row) and AR forcing (anomaly, lower row). We retain the resulting AR-forcing figure in the main article and include the climatology in supplementary.

REVIEWERS' COMMENTS

Reviewer #1 (Remarks to the Author):

The authors have addressed all of my concerns with the original manuscript. The revised manuscript represents a significant improvement and is ready for publication.

Reviewer #2 (Remarks to the Author):

The authors have sufficiently addressed all of my comments and concerns. Upon review of the revision I just have a couple minor points to add:

1) Lines 135-136: The authors note that the surface and ocean fluxes contribute to the variability in the Kuroshio Extension region equally, however, from Figure 5 this does not appear to be the case. While a very small 0.6 contour is found in the KE region in Figure 5f, the contours around the KE region in 5b are greater in magnitude and cover a much larger areal extent. As far as I can tell from Figure 5, KE variability (i.e. variability along 40N) is dominated by advection and vertical mixing, which is mentioned in the next sentence.

2) Figure 6a-c: The labels in the figure caption do not match the labels on the figure itself. Please fix.

3) Line 144: There is a typo where "due" is written as "du."

4) Make sure the supplementary figures are numbered in the order they are referred to. Both Figures S3 and S4 are mentioned before S2.

Reply to Reviewer 2’s Comments on “Response of Sea Surface Temperature to Atmospheric Rivers”

Dear reviewer,

Thank you for your additional comments and review of our manuscript. Your feedback helps refine the content. In this revision, we have addressed your comments on the variability as shown below. And we thank you for pointing out formatting problems in detail as well.

Reviewer #2 (Remarks to the Author):

The authors have sufficiently addressed all of my comments and concerns. Upon review of the revision I just have a couple minor points to add:

1) Lines 135-136: The authors note that the surface and ocean fluxes contribute to the variability in the Kuroshio Extension region equally, however, from Figure 5 this does not appear to be the case. While a very small 0.6 contour is found in the KE region in Figure 5f, the contours around the KE region in 5b are greater in magnitude and cover a much larger areal extent. As far as I can tell from Figure 5, KE variability (i.e. variability along 40N) is dominated by advection and vertical mixing, which is mentioned in the next sentence.

Reply: Yes, the ocean contributes to the variability more than the atmosphere (although does not dominate the overall variability). We have rewritten the sentence as follows: “The ocean response (Figure 5b) contributes more variability in the Kuroshio Extension region than the surface fluxes (Figure 5f), highlighting the role of mesoscale eddies as they enhance the air-sea fluxes when AR passes above [citation Liu et al. 2021].”

2) Figure 6a-c: The labels in the figure caption do not match the labels on the figure itself. Please fix.

Reply: We have fixed this issue.

3) Line 144: There is a typo where "due" is written as "du."

Reply: We have fixed the typo.

4) Make sure the supplementary figures are numbered in the order they are referred to. Both Figures S3 and S4 are mentioned before S2.

Reply: We have put the correct order now.
